# The discovery of the Amazonian tree flora with an updated checklist of all known tree taxa

**DOI:** 10.1038/srep29549

**Published:** 2016-07-13

**Authors:** Hans ter Steege, Rens W. Vaessen, Dairon Cárdenas-López, Daniel Sabatier, Alexandre Antonelli, Sylvia Mota de Oliveira, Nigel C. A. Pitman, Peter Møller Jørgensen, Rafael P. Salomão

**Affiliations:** 1 Naturalis Biodiversity Center, Vondellaan 55, Postbus 9517, 2300 RA Leiden, The Netherlands; 2 Coordenação de Botânica, Museu Paraense Emílio Goeldi, Av. Magalhães Barata 376, C.P. 399, Belém, PA 66040–170, Brazil; 3 Herbario Amazónico Colombiano, Instituto SINCHI, Calle 20 No 5-44, Bogotá, DF, Colombia; 4 Institut de Recherche pour le Développement (IRD, UMR AMAP), TA A-51/PS2, Bd. de la Lironde, 34398 Montpellier cedex 5, France; 5 Department of Biological and Environmental Sciences, University of Gothenburg, Box 461, SE-405 30 Göteborg, Sweden; 6 Gothenburg Botanical Garden, Carl Skottsbergs gata 22A, SE-413 19, Göteborg, Sweden; 7 Science and Education, The Field Museum, 1400 S. Lake Shore Drive, Chicago, IL 60605–2496, USA; 8 Nicholas School of the Environment, Duke University, Durham, North Carolina 27705, USA; 9 Missouri Botanical Garden, P.O. Box 299, St. Louis, Missouri 63166-0299, USA

## Abstract

Amazonia is the most biodiverse rainforest on Earth, and the debate over how many tree species grow there remains contentious. Here we provide a checklist of all tree species collected to date, and describe spatial and temporal trends in data accumulation. We report 530,025 unique collections of trees in Amazonia, dating between 1707 and 2015, for a total of 11,676 species in 1225 genera and 140 families. These figures support recent estimates of 16,000 total Amazonian tree species based on ecological plot data from the Amazonian Tree Diversity Network. Botanical collection in Amazonia is characterized by three major peaks, centred around 1840, 1920, and 1980, which are associated with flora projects and the establishment of inventory plots. Most collections were made in the 20th century. The number of collections has increased exponentially, but shows a slowdown in the last two decades. We find that a species’ range size is a better predictor of the number of times it has been collected than the species’ estimated basin-wide population size. Finding, describing, and documenting the distribution of the remaining species will require coordinated efforts at under-collected sites.

The forests and savannahs of the Amazon basin and Guiana Shield (here Amazonia) arguably hold the greatest biodiversity on Earth: an estimated 1300 species of birds, 427 species of mammals and 50,000 species of seed plants^1^,^2^. Whereas the collection record for birds and mammals may be relatively complete and the estimates for those taxa relatively precise, plants in Amazonia remain hugely under-collected^3^,^4^. Indeed, 300 years of plant collecting in Amazonia have resulted in a modern-day density of only 10 collections/100 km^2 ^5,6. Feeley and Silman^7^ suggested that the lack of data is so strong that there exists a ‘*gaping data void’ such that many species and many habitats remain… functionally invisible for most studies”.* Data is not only scarce; it is also often unavailable^3^,^5^, and biased spatially and taxonomically^8^,^9^,^10^.

As a result, the number of tree species in Amazonia remains unknown. No checklist of all Amazonian trees has ever been compiled, and estimates of the size of the Amazonian tree flora are hotly contested because data is scant and extrapolation techniques are abundant. Recently, ter Steege and colleagues estimated the number of Amazonian tree species that exceed 10 cm diameter at breast height (DBH) at ~16,000, by fitting a logseries curve to population estimates of ~5000 species identified in 1170 inventory plots across Amazonia^11^. That hypothesis has been embraced as plausible by some authors^12^ but criticized by others^13^,^14^. Non-parametric estimates, that were suggested by an anonymous reviewer of the above paper, predict considerably lower plant diversity: 6000–7000 species in the case of Amazonian trees^11^.

To provide a stronger empirical foundation for this debate, here we provide a preliminary checklist of all valid tree species collected to date in Amazonia. We then analyse the list to explore why some Amazonian tree species are more frequent in herbaria and other floristic datasets than others. Does a species’ frequency in these datasets reflect its abundance and range size, the date it was first discovered in Amazonia, the spatial pattern of Amazonian exploration, or some combination of those factors? Answering these questions will allow us to discuss the dynamic behind the discovery of new species and consequently the best approach to complete the inventory of the Amazonian tree flora.

## Results

### Diversity and collection density

The checklist contains 11,187 valid species names (Appendix S1) from 530,025 unique collections of trees made in Amazonia over the last three centuries, from 1707 to 2015, as well as 489 valid species names known to occur in Amazonia but lacking occurrence record data. The total number of species in the checklist is 11,676.

**Figure 1:**
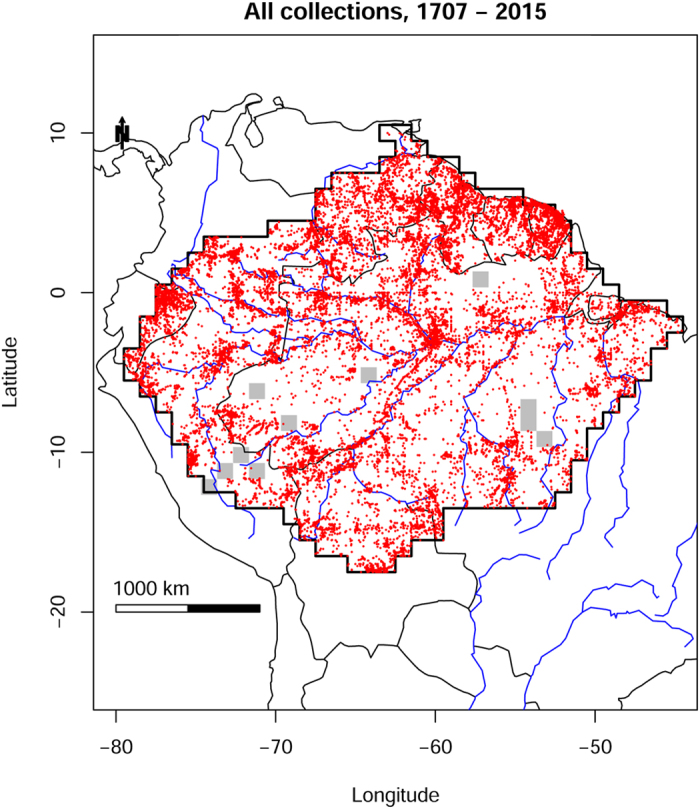
Collection localities of herbarium specimens of Amazonian trees, collected between 1707 and 2015, for which geographical coordinates were available and considered reliable. Grey squares are 1-degree grid cells that contain no collections. Map created with custom R script. Base map source (country.shp, rivers.shp): ESRI (http://www.esri.com/data/basemaps, © Esri, DeLorme Publishing Company).

Average collection density (530,025 specimens from an area of 5.6 million km^2^) is just under 10 per 100 km^2^. Collections are spread across Amazonia, but concentrations of collecting effort are clearly visible in the Guianas, around large Amazonian cities (e.g. Belém, Manaus), and along major rivers and roads (Fig. 1). Thus, while a 1-degree grid cell (111 × 111 km^2^) in Amazonia contains ~950 collections on average, 31 grid cells contain more than 2500. The grid cell in which Manaus is located has 24,598 collections (Fig. S1). At the poorly collected end of the spectrum, half of all grid cells contain fewer than 200 collections. Eleven grid cells contain no tree collections at all, and together these cover an area of approximately 320,000 km^2^.

The vast majority of the Amazonian tree species in our preliminary checklist (96%) come from our initial collections database (GBIF, SpeciesLink, INPA, MG, and COAH). Roughly 41% of all Amazonian tree species (4657) have been recorded in ATDN tree plots to date (Appendix S1) and 104 of these were not found in the herbarium databases we examined. Additional species (i.e., species not in the initial collections dataset or ATDN) were added from checklists, catalogues, and IPNI (385). Correcting all names of Annonaceae with Maas *et al.*^15^ resulted in 22 changes in the 498 names of Amazonian Annonaceae (down to 481). Five of these were simply a change in name, while 17 were the merging of two species into one new synonymy. The latest *Guatteria* treatment made more of an impact: 62 species were put in synonymy of 15 species (10 into *G. hirsuta* and 26! into *G. punctata*) and 10 new Amazonian species were described^16^.

### Taxonomic patterns

The 11,676 tree species in the checklist belong to 140 families and 1225 genera. Fabaceae was, as expected, the family with the largest number of accepted tree species (1611), followed by Rubiaceae (1058), Melastomataceae (624), Myrtaceae (606), Lauraceae (566), Annonaceae (480), Euphorbiaceae (351), Chrysobalanaceae (312), Malvaceae (304), and Sapotaceae (278). These 10 families accounted for 53% of all tree species (Appendix S3). Fifteen families were represented by a single species (Appendix S3). The ten most speciose genera were *Miconia* (307 species), *Eugenia* (209), *Ocotea* (190), *Inga* (184), *Psychotria* (182), *Myrcia* (173), *Licania* (159), *Pouteria* (154), *Solanum* (147), and *Swartzia* (144), which together accounted for 16% of all species (Appendix S3). More than a third of all genera (451 out of 1225) were monospecific in the Amazonian tree collections.

### Collection frequency, estimated population size, and range size

The tree species with the highest number of collections was *Hirtella racemosa* (Chrysobalanaceae), with 1623 collections, followed by *Tapirira guianensis, Eschweilera coriacea, Licania heteromorpha, Siparuna guianensis, Cordia nodosa, Virola elongata,* and *Micropholis guyanensis*. Fifty percent of all collections were accounted for by the 745 most collected species; these represent 6.7% of all tree species in the collections dataset.

**Figure 2:**
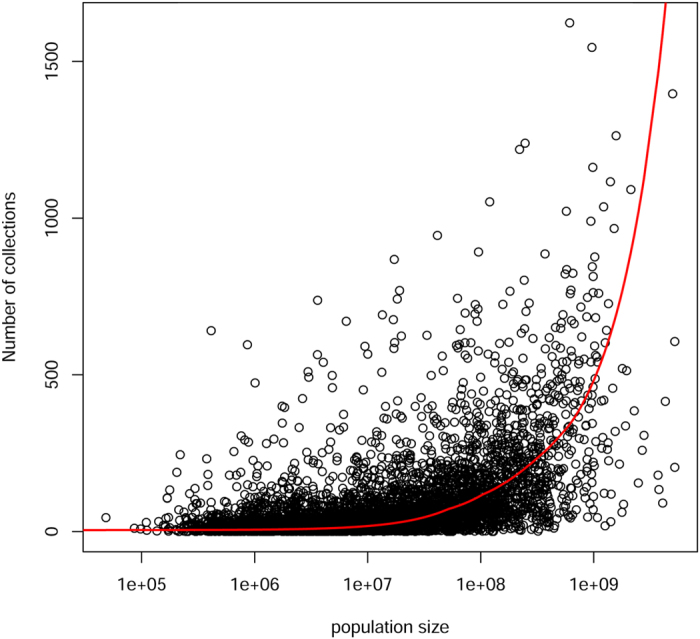
The effect of estimated basin-wide population size on the number of specimens in herbarium databases, for 11,187 Amazonian tree species. Note that x-axis is log-scale. Red line: local weighted regression (loess) through all data.

**Figure 3:**
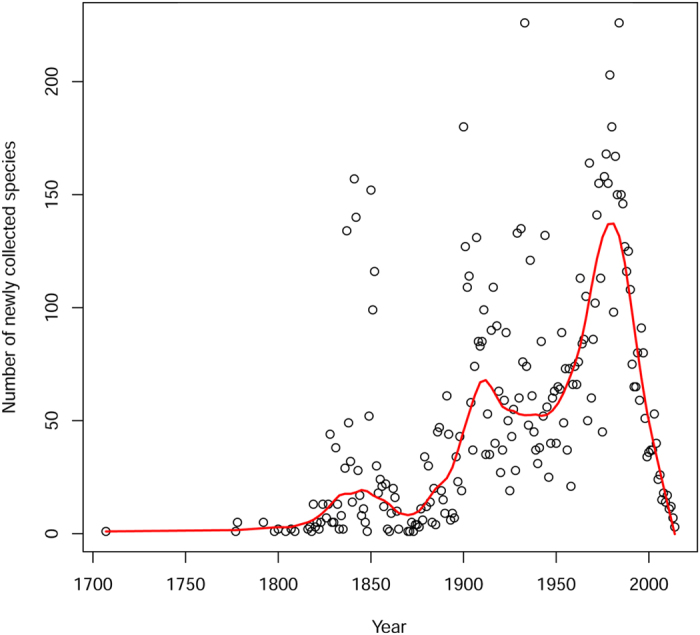
The number of first-time collections of Amazonian tree species each year between 1707 and 2015. The red line (loess regression) suggests 3 peaks in discovery. The decline after 1980 is partly the effect of decreasing efficiency in the rate of new species per number of collections (see Fig. 6) and partly the effect of the lag time between the collection of new species, the description of new species, and the appearance of new species in herbarium databases.

Estimated population size had a moderate effect on the number of collections per species (Fig. 2, linear regression R^2^ = 22%, p ≪ 0.001). Only 139 of the 227 hyperdominants, sensu ter Steege *et al.*^11^, were among the most frequently collected species that accounted for 50% of all collections. Among species with population sizes ranging from 10^5^ to 10^7^ individuals, the mean number of collections was nearly constant at 14 collections per species. For species with estimated populations between 1 and 10^6^ individuals^11^, the mean number of collections was also around 14 (data not shown, see Appendix S1). Among species with population sizes over 10^8^, the mean number of collections rose sharply to 200 collections per species, for a final average of close to 700 for species with populations over 10^9^ individuals. There was a similarly small effect of rank population size on rank number of collections (Fig. S2), with the former explaining 35% of the variation in the latter. Population size had no noticeable correlation with the date a species was first collected (Fig. S3). Species discovery has increased through time starting in 1800 (Fig. 3), with peaks in discovery centred around 1820–1850, 1900–1920, and a large peak around 1975–1985.

**Figure 4:**
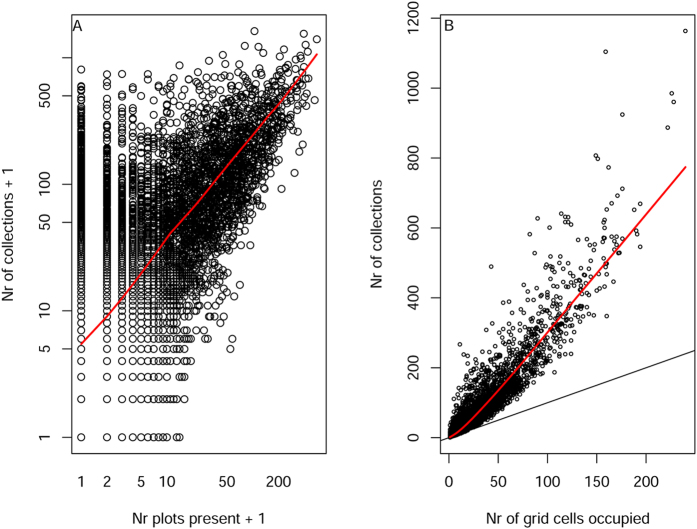
(**A**) The number of ATDN plots in which a species is present^11^, a proxy for range size across Amazonia, as a predictor for the number of collections. (**B**) The number of grid cells in which a species has been collected, a non-independent proxy for range size, as a predictor for the number of collections.

Range size, as defined by the number of plots in which a species has been found, had a moderately strong effect on the number of collections (Fig. 4A, linear regression: R^2^ = 44%, p ≪ 0.001). Species that were found in 100 plots or more had on average more than ten times the number of collections than species observed in fewer than ten plots. However, some species only observed in 1–5 plots had close to 500 collections. The number of grid cells in which a species has been collected, another measure for its range size (Fig. 4B), is strongly correlated with the number of collections (R^2^ = 88%, p ≪ 0.001). With a slope of 3, on average species are collected 3 times per grid cell in which they have been collected. After accounting for the number of grid cells in which a species occurs, neither the number of plots nor the population of the species adds more than 1% explained variation to the number of collections.

**Figure 5:**
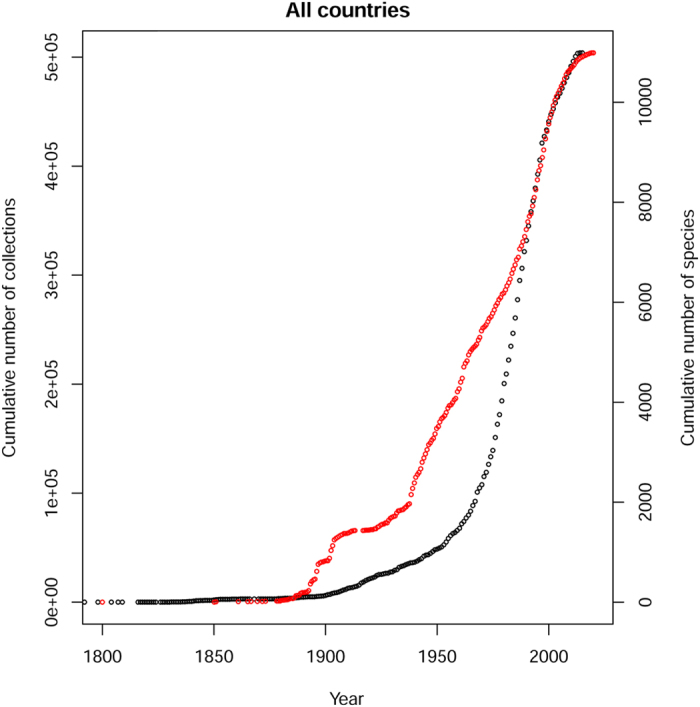
Cumulative number of tree species specimens collected in Amazonia from 1800 to 2015 (Black line, left y-axis). Cumulative number of tree species collected in Amazonia from 1800 to 2015 (red line, right y-axis).

**Figure 6:**
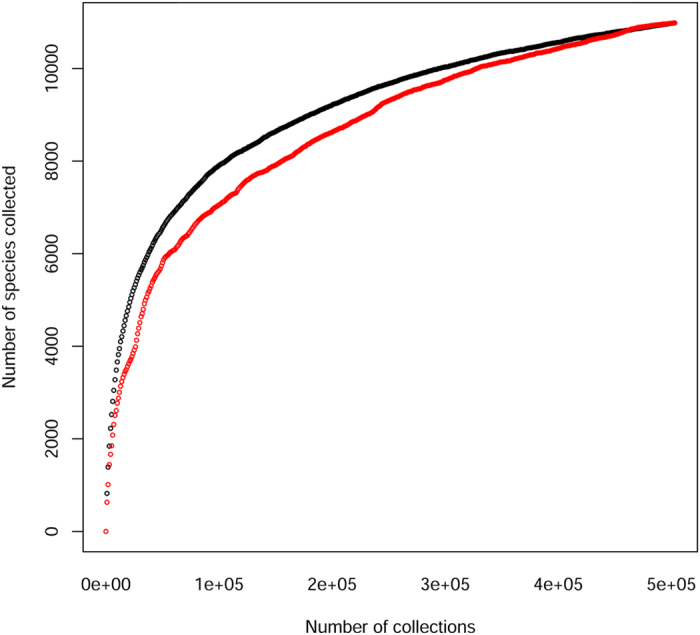
The number of species as a function of the number of collections. Black line: data randomized with regard to year of collection. Red line: data in order of date collected.

We found only a few thousand collection records (5696) from the period before 1900, after which the average collection rate increased to 839 specimens/year. After 1960, the collection rate rose again sharply to 10,380 collections/year. There have been fewer new collections in the last 10 years (4727/year from 2000 to 2010 and 2636/year for the last five years (Fig. 5). The rate of tree species discovery in Amazonia has risen faster than the rate of collections (Fig. 5), with a rate of 35 newly collected tree species per year between 1820 and 1850. This rate slowed considerably (13 new species per year) between 1850 and 1875, after which it rose to an average of 84 newly collected tree species/year from 1900 to 2000, peaking from 1975 to 1985 with an average of 164 newly collected species/year. The randomized species-collection curve (Fig. 6) suggests that more tree species will be found in Amazonia if substantial collecting effort continues (see discussion). But the number of collections required to find a new species has increased substantially over the last 300 years, from a few collections per new species in the 1800 s to ~300 collections for a new species now (Fig. S4).

### A historical perspective on the discovery of the Amazonian tree flora

#### 1700

Our data suggests that the earliest collections of Amazonian trees were mainly made in the three Guianas (Figs S5 and S6). More than half of the collections (3562) before 1900 came from this area. By area the Guianas are still the best collected countries in Amazonia by far (Table 1). Jean Baptiste Christian Fusée Aublet may have been the first serious collector in the region, responsible for the first collections of 211 tree species (Appendix S1), including many type specimens from French Guiana (1762–1764). Based on his collections he published the ‘*Histoire des plantes de la Guiane françoise*’^17^.

In the same period, practically all of the other Amazonian countries were botanically explored during the expeditions of Joseph de Jussieu (1704–1779). Although he made many other scientific collections in addition to plants, Jussieu was the first naturalist to collect plant specimens in Ecuador, Peru, and Bolivia, as a participant of the French Geodesic expedition from 1735 to 1743^18^, together with the geographer and naturalist Charles Marie de La Condamine. After the French Geodesic expedition, de La Condamine, together with Louis Godin, Pierre Bouguer and the Spaniards Jorge Juan and Antonio de Ulloa, carried out an expedition from Quito to the Atlantic Ocean, through Ecuador, Colombia, and Brazil (and from there to France). This is considered to be the beginning of the great travels of European scientists in Amazonia, and led to the description of the quinine tree (*Cinchona officinalis*) and the rubber tree (*Hevea* spp.). Also Alexandre Rodrigues Ferreira (1758–1815), merchant from Bahia, traveled through Amazonia to Mato Grosso (1783–1792) and collected many plants, now located at Paris and LISU.

Despite being visited by Jussieu, Bolivia was – and still is – one of the least explored western Amazonian countries^19^ (Table 1). Some other early botanical exploration in Bolivia, of minor impact, include the French botanist Louis Feuillée (1660–1732) and the Austro-Hungarian naturalist and explorer Thaddaus Peregrinus Xaverius Haenke (1761–1816). Another pioneering expedition worth mention, although not centred in Amazonia, was that of Hipólito Ruiz, who travelled to Peru in 1777–1784 and later to Chile, writing the ‘*Florae Peruvianae et Chilensis prodromus*’.

The only country that was not visited by the two main collecting efforts of the 18^th^ century (Aublet and Jussieu) was Venezuela. The first documented expedition in the country was the ‘*Comisión de Límites*’ in 1755. The main botanist on the trip, Pehr Loefling, a student of Linnaeus, fell seriously ill and died and his plants were lost^20^. Later, Alexander von Humboldt and Aimé Bonpland collected in Venezuelan Guayana in 1800 and their excellent collections were the basis for further studies in the area. They travelled to the Orinoco and the Rio Negro, making important botanical collections in Venezuela, Brazil, and Colombia.

#### 1800s

After 1800 many more naturalists were active in Amazonia. In the Guianas, there were two increases in collecting between 1804 and 1853 (Fig. S6B,C). The first peak occurred in 1837 due to the collecting effort of Frederick Louis Splitberger in Suriname, the second to collections by Robert and Richard Schomburgk, who travelled extensively between Guyana and Venezuela from 1835 to 1844 and published considerably^21^,^22^.

Richard Spruce (1817–1893) spent almost seven years in Ecuador, from 1857 to 1863, but collected mainly in the highlands and left for Peru in 1863^18^. The most representative work of Spruce for the Amazonian Flora is the result of his collections in Venezuela and Brazil. He made over 800 botanical collections, many of which were new to science^20^. Apart from the general botanical collections, his study of Amazonian bryophytes is a benchmark in the Neotropics, with the description of hundreds of new species. Also in the first half of the 19^th^ century, Juan Isern y Batlló (1825–1866) and companions on the Royal Spanish Pacific expedition left Ecuador through the Amazonian lowlands and travelled down the Amazon river to Manaus, Belém, and finally Pernambuco^18^.

But the expedition that had the greatest impact on knowledge of the Amazonian tree flora in the 19^th^ century was probably that of Carl Friedrich Philipp von Martius (1794–1868). With zoologist Johann Baptist von Spix he travelled four years (1817–1821) from Rio de Janeiro to São Paulo (in south-eastern Brazil) to Belém, and from there up the Amazon River by canoe. Overall, he and his team travelled 10,000 km and made 3,541 collections of birds, insects, and other animals, and 25,000–30,000 herbarium collections^23^,^24^, which contain ca. 7,300 plant species, and are housed at the Botanische Staatssammlung München. Perhaps even more importantly, von Martius initiated the ‘*Flora Brasiliensis*’ project, containing taxonomic treatments of 22,767 species, mostly Brazilian angiosperms. He completed 46 of the 130 volumes before his death in 1868, with the monograph being completed in 1906 (http://florabrasiliensis.cria.org.br/).

#### 1900s

In the 20^th^ century, the number of tree specimen collections and the number of tree species recorded for Amazonia increased by two orders of magnitude (Fig. 5). Collection effort also varied among countries (Fig. S6), because efforts became much more divided. While earlier efforts were concentrated in large expeditions that crossed country borders, the increased sovereignty of countries after independence from colonial rule led to localized and national programs of botanical exploration. Furthermore, the start of several flora projects intensified the study of the region.

Below a brief account of the collecting history of the 20^th^ century for each of the Amazonian countries is given in order of collecting intensity. Table S1 lists the most important collectors for this period by country.

#### French Guiana

French Guiana is the best collected country by area in Amazonia, with a collecting density of 75 collections/100 km^2^ (Table 1). The cumulative number of collections increases sharply in the second half of the century (Fig. S6A); this is related to the creation of the herbarium in Cayenne by Roelof Oldeman, and the consecutive collecting efforts of Oldeman’s team and Jean-Jaques de Granville, widely spread over French Guiana, and of Scott Mori in Saul^21^. Most botanists were involved in both plot inventories and traditional collecting, making it difficult to disentangle their relative contributions. Nearly 265 ha of forest botanical inventories have been carried out in French Guiana since 1980, and these have added 350 new tree species for the country. During the same time, only ~60 tree species new to French Guiana were collected outside the plots.

#### Suriname

Up to 1953 fieldwork of botanists collecting under the ‘Boschwezen’ number series, mainly in the Zanderij, Sectie O, and Brownsberg areas and along the Nickerie River, added greatly to the number of collections in Suriname (Fig. S6B). In the same period collections intensified with the start of the ‘*Flora of Suriname*’^21^. Jan Lindeman added greatly to the collections of trees in Suriname and produced one of the first tree guides of the region^25^.

#### Guyana

In the first half of the century, tree collections increased with large contributions from Noel Yvri Sandwith around the Mazaruni Station and from Albert Charles Smith around the Cuyuwini landing and in the Rupununi Savannah (Fig. S6C). The continued field trip and collections of the Forest Department led to a great increase in the knowledge of forest types and their dominant tree species^26^. A second increase, after the 1980s, reflected a change in collecting strategy, with large expeditions targeting specific geographical areas within the scope of projects such as the ‘*Flora of the Guianas*’, started in 1984^21^. At this moment 3744 species of trees have been collected in the Guianas (Appendix S2, Table 1) and using the estimate of expected species richness for the Guianas of 4581 species^11^, 82% of the tree species have been collected locally.

#### Ecuador

The number of species increased sharply in the 1950s and collecting intensified in the 1970s (Fig. S6D) with the start of the ‘*Flora of Ecuador*’ in 1973^27^. Jørgensen^18^ estimated that 50,000 plant collections were made in Ecuador prior to 1900, while ten times that number were made in the 20^th^ century. Collecting in Ecuador initially focused on the Pacific lowlands; the Amazonian lowlands were explored much later, most notably by a group of botanists associated with the National Herbarium (David Neill, Walter Palacios, Carlos Cerón, Alberto Dik). By 1993 the total number of plant collections in Ecuadorian Amazonia was estimated at 61,000^27^. In our dataset the total number of tree collections for Amazonian Ecuador is 16,202 (Appendix S2, Table S1). While this makes the region the fourth-best collected in Amazonia (Table 1), our data suggest that much more collecting will be needed to record the full tree flora. With 2366 tree species collected to date, just 35% of the estimated total number of tree species in the Ecuadorian Amazon have been recorded there.

#### Colombia

Between 1940 and 1950 botanical collecting increased (Fig. S6E). Richard Evans Schultes made important collections in the department of Vaupés, adding a great number of new species and new records to the Colombian flora. José Cuatrecasas visited the forests of the Orteguaza River (Caquetá), the Vaupés River, the Serranía de la Macarena (Meta) and the Serranía de la Lindosa (Guaviare), where he collected valuable botanical material. Between 1972 and 1979 the Project Radargramétrico del Amazonas (PRORADAM) generated important collections of timber species in the Colombian Amazon. In the 1990s many collections were made for ecological studies of Amazonian forests. The Herbario Amazónico Colombiano (COAH) del Instituto SINCHI, created in 1983 to focus on the country’s Amazonian flora, now holds ~95,000 specimens and 7,700 species of vascular plants. A new catalogue of all plants of Colombia was published in 2015^28^. In our dataset the total number of tree collections for Amazonian Colombia is 35,277 (Appendix S2, Table 1). With 3511 tree species collected, 35% of the estimated number of tree species have been collected in the Colombian Amazon.

#### Brazil

Brazil is the country with the highest number of collections (278,165) for an average density 6.8 collections/100 km^2^. The first peak of collections occurred very early in the century (Fig. S6F), when the herbarium of the Museu Paraense Emílio Goeldi em Belém (MG) was created. Later, many Amazonian expeditions organized by the Rio de Janeiro Botanical Garden contributed to the increase of collections thanks to Jacques Huber (1867–1914), João Geraldo Kuhlmann (1882–1958), and especially Adolpho Ducke (1876–1959)^29^. Nelson A. Rosa recorded more than 6,000 collections in Amazonia and João Murça Pires (1946–1962) collected in Amazonas and eastern Amazonia (close to 5,000 collections in the data used here) and also established the first permanent plots in upland forests^30^. George Alexander Black (1910–1957) collected all over Amazonia^31^. Black also established plots and made “*attempts to estimate species diversity and population density of trees in Amazonian forests*”^32^. Ghillean Prance is arguably the collector who made the most collections of trees in Amazonia (>20,000 in the data presented here). Much new material was collected during the ‘*Projeto Flora Amazonica*’, which increased the number of collections in Amazonian herbaria by about 50%^33^. Also, the start of ‘*Flora Neotropica*’ in 1967 helped spur an increase in collections and the discovery of new species in Brazilian Amazonia, as was the case with the monographs of Chrysobalanaceae and Lecythidaceae by Prance^6^. In our dataset the total number of tree collections for Amazonian Brazil is 278,165 (Appendix S2, Table 1). With 7696 tree species collected, 61% of the estimated number of tree species have been collected in the Brazilian Amazon.

#### Peru

Twentieth-century botany in Peru was initiated by the work of Augusto Weberbauer and Ignatz Urban, which led to an immediate increase in the number of species^19^ (Fig. S6F). The ‘*Flora of Peru*’ project was started in 1922 by Francis Macbride, who added greatly to Peruvian collections himself and attracted many other collectors. Specimens generated from this project provided the Field Museum herbarium (F) with one of the world’s best overall collections of Peruvian plants; however, most of those collections have not yet been digitized. Collectors from the Missouri Botanical Garden, especially Alwyn Gentry and Rodolfo Vásquez, also contributed large numbers of Amazonian tree collections in the period 1970–2000. In 1993 another major step was completed with the ‘*Catalogue of the flowering plants and gymnosperms of Perú*’^34^. In our dataset the total number of tree collections for Amazonian Peru is 39,851 (Appendix S2, 1). With 4422 tree species collected, 47% of the estimated number of tree species have been collected in the Peruvian Amazon.

#### Venezuela

Botanical collecting picked up in the 1940s and then increased steeply in the 1970s (Fig. S6G), yet Huber^20^ considers lowland Venezuela to still be severely under-collected. The ‘*Flora of the Venezuelan Guayana*’, initiated by Julian Steyermark in the early 1980s and completed under the guidance of Paul Berry, Kay Yatskievych, and Bruce Holst, has helped the latest increase in the cumulative number of species. In our dataset the total number of tree collections for Amazonian Venezuela is 16,356 (Appendix S2, 1). With 3189 tree species collected, 49% of the estimated number of tree species has been collected in the Venezuelan Amazon.

#### Bolivia

With just 2.4 tree collections per 100 km^2^, Bolivia is by far the most under-collected Amazonian country for trees (Table 1). Remarkably, for every 1 tree specimen collected per unit area in Bolivia, 31 tree specimens have been collected in French Guiana (the best-collected country). This partly reflects a much later start in botanical exploration. At the beginning of the 20^th^ century, four independent projects were undertaken but the flora of Bolivia received virtually no study between the 1920s and 1946^35^. In our dataset the total number of tree collections for Amazonian Bolivia is 11,721 (Appendix S2, 1). With 2404 tree species collected, 40% of the estimated number of tree species has been collected in the Bolivian Amazon.

## Discussion

Nearly 12,000 tree species have been collected or observed in Amazonia to date. This makes the estimate of 16,000 total tree species for Amazonia by ter Steege *et al.*^11^ seem entirely plausible, and supports their conclusion that non-parametric extrapolation methods are not very suitable for large areas with low sampling intensities (see also^36^,^37^,^38^). We acknowledge that the estimate of 16,000 remains speculative and is based on a debated sampling theory^39^, but the logseries has not only been demonstrated empirically in a multiple of studies, but also derived by theoretical frameworks such as Neutral Theory^40^, the application of Quantum Field Theory for Biogeography^41^, and the Theory of Maximum Entropy in Ecology^42^.

Trees have not been collected randomly across Amazonia with regard to time, space, or species identity (Fig. 1, Fig. S5). This is a well-known bias, which is understood to reduce the value of collection databases for describing patterns of diversity and planning conservation^6^,^43^,^44^,^45^. What we were surprised to find is that the estimated population size of a species in Amazonia is a poor predictor of the number of times it has been collected, and of the date it was first collected in Amazonia (Figs 2 and S3). This appears to be the result of how trees have been collected. Collecting started from a limited number of points, where many or perhaps most species, both common and rare, were collected. The fact that many collectors tend to maximize the number of species for a given number of collections^44^ causes a strong disconnect between local abundance and local number of collections of tree species (Fig. 2). When new areas are visited the same pattern repeats itself. The data of one of the first Amazonian collectors, Aublet, is a good example of the efficiency of collectors. Aublet collected 211 tree species in French Guiana (Appendix S1). These species differ in estimated population size by 5 orders of magnitude and 12 of them have not been encountered in tree inventory plots in French Guiana. Because in most areas common as well as rare species are collected, range size affects the number of collections much more than abundance, since wide-ranging species are likely to be collected in many local inventories. This effect is so strong that after accounting for range size, population size did not add a significant amount of explained variation.

That plant collectors have recorded nearly 12,000 species with a collection effort of fewer than 10 collections per 100 km^2^ likewise reflects their high efficiency at capturing rare taxa^44^. Ter Steege *et al.*^11^ estimated that 11,000 species of Amazonian trees have populations of ≤1 million individuals, and that the rarest 5000 species are represented by ≤1000 individuals. The chance of randomly collecting a tree belonging to a species with ≤1 million individuals is 10^6^/~4 * 10^11^ = 2.5 * 10^−6^. For species with populations of ≤100 individuals, the probability drops to 2.5 * 10^−9^. And these very low probabilities significantly overestimate the probability that a collector who finds the tree will collect it, since most collectors will only collect specimens if they have flowers or fruits (see below). It would seem that finding a needle in a haystack might be easier. Yet, nearly 7000 of these 11,000 rare species have been recorded to date, and are found in the herbarium record.

The species accumulation curve by collections (Fig. 6) makes it clear that more species will be found with additional collection effort. New species are also expected to arise from careful inspection of herbarium material^16^,^46^,^47^, especially as more material is made available online through platforms such as GBIF and SpeciesLink.

Our list of tree species contains many species that rarely exceed 10 cm DBH. These species are necessarily rare in tree inventory plots (where 10 cm is the diameter cut-off) but they are not necessarily rare in Amazonia. A good example is *Hirtella racemosa*, a small tree which ranks #1 in number of collections (Appendix S1) but only #133 in estimated population size based on the ATDN plots^11^, appendix S1. *Tococa guianensis* is another example, ranking #25 in number of collections and occasionally reaching 8 m in height in Amazonia (e.g., Douglas C. Daly 7148) but mostly smaller and never identified in the ATDN plots. The opposite is also true. Palms are extremely abundant in the ATDN plots, accounting for 6 of the ten tree species with the largest estimated populations^11^, but are much less collected due to the enormous specimens they generate and the amount of work that this requires. Other factors that may influence the frequency of collecting are the phenology of species with regard to the timing of collecting trips, which are most often in dry periods^21^,^22^; irregular flowering and fruiting, which means that some species in tree plots are not found in fertile state despite frequent visits over 10 consecutive years (D. Sabatier, personal observation); the fact that it is easier to collect understory trees than canopy trees; and the varying (in)conspicuousness of reproductive structures in different taxa^48^. Additional factors may include temporal fluctuations in taxonomic research funding, inventory campaigns in connection with new protected areas, environmental assessments, and floristic projects.

Finally, very rare species may actually be species common elsewhere; species from the Atlantic forest, Caatinga and Andes have all been collected rarely in Amazonia (Fig. S7). This may suggest that singleton species are mainly found on the edges of Amazonia. That is, however, not the case – they are more often found in rich areas with high collecting intensity (e.g. the area surrounding Manaus and French Guiana [Fig. S8]).

Data from public repositories contain various errors^8^. In the data used here at least five types of errors can be found: taxonomy, life-form, identification, location, and incomplete digitization. The third and fourth errors occur at the first stage of data collecting (i.e., at the individual stem level) and are very difficult to find and correct after data have been aggregated.

1. Taxonomy: This error is independent of data collection. New taxonomic treatments of families and genera will move species currently on our checklist into synonymy. The impact on our list was relatively small in the case of a nomenclatural treatment of all Annonaceae (~3%, see above) but much greater in the full systematic treatment of the genus *Guatteria* (of the same family)^16^. Further study of specimens will also add to the checklist via the formal description of new species, as shown by the several new species added from IPNI but not yet present in the herbarium databases and the 10 new species of *Guatteria* described in^16^. The taxonomy of Amazonian tree families and genera is in a continuous state of flux and improving molecular techniques may cause great changes in the current classification and number of species, genera, and families. Recent phylogenetic analyses of Chrysobalanaceae^49^, Protieae (Burseraceae)^50^, and Lecythidaceae^51^,^52^ show that our view of large Amazonian tree genera and families will change dramatically in the near future. Work on other large families is also ongoing (e.g. Annonaceae – Paul Maas, pers. comm.; Sapotaceae, Lauraceae - Jerome Chave, pers. comm.).
2. Life form: When we were unsure whether a given species qualified as a tree by our definition, our main references were a recent checklist of South American trees^53^ and the ATDN species list^11^. Other sources included the ‘Tree flora of the Neotropical region’^54^, tree checklists from TROPICOS (http://mobot.mobot.org/W3T/Search/vast.html) and we checked all these new species on their protologue and on herbarium labels for their habit. Because life form can be fluid within a species, it is possible that our checklist includes a small number of species that are primarily shrubs or lianas. We believe the amount of non-tree species in our list is quite small.
3. Species identifications: An unknown but significant number of specimens in our collections dataset are incorrectly identified^55^,^56^. Incorrect identifications in herbaria have a tendency to snowball, as botanists match unidentified specimens to wrongly identified material. In some cases, duplicates of the same collection may receive different names in different herbaria. In public repository data such errors are difficult to check and may have slightly increased our species list.
4. Location: Many digitized collections contain location errors, many of which we were able to correct. Errors in location data lead to errors in species richness prediction and tend to overestimate local richness as species are wrongly positioned outside their actual range. With many species these errors add up^8^. As we constructed one large list and checked quite substantially for errors in geography, removing data with dubious location, we believe this is not a major problem in our list.
5. Incomplete digitization: Many large herbaria have not digitized their collections and deposited the data in GBIF, and their collections are only partially represented in our checklist. For example, most collections from the major expeditions outside the Guianas before 1900 were deposited in P and M (Paris and München), and records from these two herbaria were not present in GBIF when we downloaded our data. This bias might potentially explain why our dataset shows that early collecting was focused on the three Guianas (Figs S5 and S6). However, the patterns and species counts reported here for the period before 1900 are likely robust to this potential shortcoming, since most historical collectors made numerous duplicates that were widely distributed among or sold to herbaria and private collectors. Collections from the 20^th^ century are probably less vulnerable to this type of bias, since they have more complete label data, label data in English, and were collected under more established herbarium practices that make digitization easier and more likely (including the sharing of duplicates with multiple herbaria).

We have shown that collecting of trees is not random in Amazonia, consistent with^44^.The non-random nature of collections has strong implications for species distribution modelling, where the collections are assumed to be a random sample of an (unknown) probability distribution. The very effective collecting strategy of botanical collectors, stratified by geographical access, violates this assumption. This suggests that species distribution models for poorly sampled regions, such as Amazonia, may not only be compromised by the low number of observations^6^ but also by the poor representativeness of their total ranges^5^,^57^.

The accumulation of numbers of collections and species has slowed in the last 1–2 decades. This may reflect a number of factors. First, it may be largely artefactual: a result of the lag time between collecting a specimen and making its data available^47^. Specimens need to be identified, inserted in herbaria, databased, and in some cases described as new species, and then the data need to be uploaded to an online aggregator (such as SpeciesLink or GBIF). The latter is done on a regular basis by most large herbaria, but not on short time intervals and not by many smaller herbaria. Many undescribed species may also be found in recent^47^ or older^46^ collections. As an example, the 10 newly described *Guatteria* species in ref. 16 were collected for the first time in Amazonia between 1943 and 1991 (Appendix S1). Second, the decline could reflect a shift from general collection and plot work to more phylogenetic research, which targets specific groups and does not lead to broad collections. Third, it may reflect a decrease in collecting expeditions due to increased difficulties in getting permits and funding. We suggest that the first option is most likely, as the lag-time is well established and the second and third options are more speculative - establishment of plots in Amazonia does not seem to show such decrease (Hans ter Steege, pers. obs.) and plant collection is done mainly by national researchers, who, in most of the countries, have permits linked to their institutes or research projects.

What are the most efficient strategies to complete the documentation of Amazonia’s tree flora? Where, how, and how fast can we collect the species of Amazonian trees that remain undescribed? A simple extrapolation of the collection-species curve (Fig. S9) suggests that if the current logarithmic relationship between the number of species and collections remains valid, at least 4.5 million unique new collections will be required to discover all 16,000 species predicted to occur in Amazonia. At the current rate of collections per year (13,500), this will take over 300 years. And although deforestation is currently lower^58^ than previously predicted^59^, further loss of forest will inevitably lead to the loss of rare, small-ranged species before they can be discovered. If deforestation were to increase to levels of the early 2000s, most of the rare - and possibly unknown - species in eastern and southern Amazonia would face threat of extinction^58^.

There is no doubt that intensifying collecting efforts in general would result in new species for science or new species records for Amazonia. But the probability that a botanist will collect a tree species not yet in our list is small and will continue to decline (see above). Intensifying collection effort therefore should not be restricted to collecting more specimens. We recommend focusing on data exchange and curation, digitization of specimens, support of taxonomic descriptions and monographs of families, in addition to field collecting that targets specific areas or taxa.

### 1) Digitize all Amazonian herbarium specimens

It has been estimated that perhaps 50% of all undescribed species are already present in natural history collections^46^. If this is true, then several hundred Amazonian tree species have been collected and deposited in herbaria but not yet ‘discovered’ or described, as was shown in the recent example of *Guatteria*^16^. Discovering those new species in herbaria is a slow process - because huge numbers of specimens remain overlooked by taxonomists. Digitization efforts combined with search engines will quickly inform taxonomists about the specimens of the focal taxon available in the different herbaria, helping the choice of material to study and facilitating the spotting of possible new species that should be studied more carefully. To resolve these problems, we recommend aggressive digitization of herbarium collections combined with data curation by specialists, such as proposed in Reflora (http://floradobrasil.jbrj.gov.br/). Naturalis’s recent advances at the Naturalis Biodiversity Center in the Netherlands may serve as a model^60^,^61^,^62^. With a grant of €13 million, and over a period of two and a half years, 4.4 million herbarium specimens (and 4.1 million other specimens) were digitized at an average price of €1.52 per specimen^61^. In three automated ‘herbarium digistreets’ 22,000–24,000 herbarium sheets were imaged per day, with a record of 35,000 on a single day. Label data was then added into a database using the digital images. The digital material is available through Naturalis’ Bioportal (http://bioportal.naturalis.nl/). A similar effort involving MNHN-Paris and IRD-Cayenne is being carried out in France as part of the e-ReColNat project (https://recolnat.org/) and will soon give higher visibility to historical Brazilian collections. This massive digitization should be coupled with equally intensive quality checking of the digital label data, ensuring quality for the geographical locations, typological correctness of the names of the species and collectors, etc.

### 2) Support and develop taxonomic and floristic expertise

Taxonomy seems a science of the past. Universities have largely abandoned natural history collections, together with the field of taxonomy^63^,^64^. In some cases this has led to a concentration of all natural history collections in one institute, as in the case of Naturalis^60^. However, unfortunately even natural history museums are focusing more and more on non-systematic research^65^. ‘*Taxonomy and systematics are NOT stamp collecting*’^66^, however, and ‘*taxonomy can justly be called the pioneering exploration of life on a little known planet*’^67^. Taxonomy is non-linear hypothesis-driven research^64^,^68^ and the hypotheses of species delimitations can only be made by taxonomists who have spent ample time studying a particular group. The inclusion of new techniques and the increase of known species have slowed this process instead of speeding it up, as more material needs to be consulted and more tests carried out^68^. As new species are found among the collections, primarily during family and genus revisions or monographs, and as these can only reliably be assessed by experts, we need to maintain and extend taxonomic expertise and train and support young taxonomists so that they can work for years on large and difficult groups. If new identifications can easily be passed on to the large data repositories^8^, this would also improve the quality of digital information. The importance of flora projects in this process cannot be overstressed.

### 3) Accelerate and facilitate information exchange on Amazonian trees

All taxonomic literature on Amazonian trees (and in general) should be available online. Ideally all floras, revisions and monographs should be produced as e-documents with a standard format that can be updated easily. Existing alternatives are for example the Lecythidaceae pages of S.A. Mori (http://sweetgum.nybg.org/lp/) or the Scratchpad-based Lauraceae pages (http://lauraceae.myspecies.info/). All new descriptions should be online as fast as possible, either incorporated in the online e-monographs and e-floras or by being published in e-journals that also provide data exchange with e.g. IPNI^69^. Wen *et al.*^62^ provide an outline for the workflow of e-monographs. It would be an asset for the Amazonian tree flora if ‘Flora of Ecuador’, ‘Flora of the Guianas’, ‘Flora Neotropica,’ and similar floras were online with electronic keys, links to collections, and image databases. Such floras democratize floristic and taxonomic information and are updatable ‘on the fly’. It should be noted that, while e-floras and e-monographs will speed access to floristic and systematic information, it will not make taxonomic work much faster (see point 2).

We have started to amass all information known on Amazonian tree species. Based on the checklist in this paper, we created a platform (http://amazoniantreeflora.myspecies.info) where descriptions, images, DNA barcodes (GenBank, BOLD, new sequences) can be added by a growing and open community of Amazonian tree specialists. Ideally, we will assemble all collected species, all unique herbarium specimens, all species of the plots and we will have them sequenced. Identification tools and high-quality phylogenies of Amazonian tree species are also high priorities.

To aid in the identification of unidentified stems in the tree plots some of us (Daniel Sabatier and Julien Engel) are developing a platform (http://atdnmorphospecies.myspecies.info/) to share and manage images and information about “morphospecies” i.e. numbered species. These are supported by specimens expected to belong to valid but locally unknown and sometimes undescribed species. The site was developed first for French Guiana, to share information among the teams working in the same area, but will be modified make the platform useful for any tree plot contributor and for any part of Amazonia.

### 4) More focus on Amazonian research

Amazonia is still largely under-collected^3^,^4^,^6^, except perhaps for the Guianas (Table 1, S1). In Brazil most research is concentrated in the southeast and south, which have 59 of the 92 Brazilian herbaria and 67% of all Brazilian collections. Brazilian Amazonia, while covering half of Brazil, has just five registered herbaria containing just 11% of the botanical collections^70^. Also, current systematic research focuses on non-Amazonian areas. Of the 2875 new Brazilian angiosperm species described from 1990 to 2006, only 20% were Amazonian. While close to 50% of the new species occurring outside Amazonia were described by Brazilian researchers, the figure for Amazonian species was only 20%. Just 0.9% of research funding from the Brazilian government between 1997 and 2002 was dedicated to Amazonia^70^.

### 5) Target geographic areas where collection effort is low and expected diversity is high

Although many new species may be found in herbaria (point 1), collecting is still needed and may be a limiting factor as in other groups^66^. Where should botanists focus their efforts, in order to avoid having to collect 4,500,000 specimens? Probably areas and with both high diversity and low collecting density. Hopkins^6^ suggested four main regions where botanical knowledge is especially weak in Amazonia, but where biodiversity is expected to be high (1. lowland Colombia, centred in the area comprising parts of the departments of Vichada, Meta, Guainia, Guaviare and Vaupes; 2. western Amazonian Brazil, within the state of Amazonas, approximately between the cities of Tefé and Envira, comprising the interfluvial region between the Rio Purus and the Rio Juruá , and extending north of the Amazon River as far as the Jaú National Park; 3. northern Amazonian Brazil, extending from north-east Amazonas State across southern Roraima and the portion of Pará State about 300 km north of the Amazon River, and including the southern extremity of Guyana; 4. south-eastern Amazonian Brazil, extending from the southeast corner of Amazonas State (headwaters of the Rio Sucunduri and middle course of the Rio Aripuanã) and southern Pará State, especially the upper reaches of the Rio Irirí and Rio Curuá.). Based on diversity (Fig. S10B), collection density (Fig. S10A, Table 1), level of botanical knowledge (Fig. S10C) and completeness of the botanical survey (Table S1), we suggest that collecting should focus on three areas: 1) the border area of Brazil and the Guianas, 2) the large interfluves of southwestern Brazilian Amazonia, and 3) the eastern slopes and foothills of the Andes (Ecuador, Peru, Bolivia), where many new species are collected on nearly every botanical expedition (Robin Foster pers. comm.). In addition to targeting specific areas, efficiency in finding the remaining species might greatly improve if collection efforts were taxon-targeted. Focusing collecting efforts specifically on species that are not identifiable in the field and ignoring common species, would increase the likelihood of finding the missing species. We also need to keep collecting more common species, however, to widen the geographic range of collections as herbarium specimens rather than published observations are the only primary data that can be checked for correctness of identification^71^. Rare species will remain difficult to find. Hopkins^6^ suggests that well executed florula projects^29^, accompanied by consistent long-term collecting will result in the discovery of many rare species. However, even for Reserva Ducke we estimate (ter Steege *et al.* in prep), that over 2600 tree species will be found, while less than 2000 have been collected to date.

### 6) Embrace new technologies

Aublet would have been surprised to learn that most botanists still search for new plant species the same way he did in 1762. If we are to locate the rarest Amazonian tree species before they go extinct, we need to incorporate new technology, experimentation, and other emerging methods that allow us to search larger areas with greater precision. Botanists may need to spend more time in the air, or in the canopy, than on the ground in the field. One example of an emerging method for large-scale plant identification is spectranomics^72^, which in one study was able to identify 85% of 1449 species in Peru based on a 20-trait spectranomic signature for leaf reflectance. If the authors can take the next step and collect those signatures from near-remote-sensing platforms, it may become possible to map large numbers of individual trees and identify undescribed trees with unknown signatures. DNA-Barcoding, phylogenetic research, near infrared spectroscopy^73^,^74^, and other techniques will also help to identify species or discover new species through an alternative way other than monographing, yet combined with taxonomic expertise. Many of these techniques require new collection methods, which is why every Amazonian tree specimen collected from this point on should be accompanied by silica gel-dried leaf samples.

### Conclusions and prospects

We have shown that recent estimates of Amazonian tree diversity based on statistical extrapolation from a logseries^11^ are fully plausible: close to 12,000 of the ~16,000 species estimated to occur in the region have been recorded to date. The vast majority of this discovery has taken place in the last century, thanks to the effort of thousands of botanists from all over the world, flora projects, collecting expeditions, and the establishment of tree inventory plots. Finding the remaining species and better documenting all species’ distributions constitute a major scientific challenge of Amazonian botany. We argue that the most time- and cost-efficient strategy to tackle this problem is to expand the collecting effort to understudied sites, followed by taxon-focused collection campaigns and the application of new technologies. The remaining work in discovering the tree diversity of Amazonia will have far-reaching effects in ecology, systematics, conservation biology and evolution. This research program will be costly but needs to be put into perspective, as proposed by Hubbell^75^: ‘*we need far better data on the geographic ranges and abundances of tropical tree species to finally put the “how many species?” question to rest. It seems to me that our priorities are misplaced. We spend many billions of dollars to look for extra-terrestrial life but far less to understand life and its distribution on our own planet*’.

## Methods

### Data sets

All plant collection records for the nine Amazonian countries available at GBIF (http://www.gbif.org; 1.5 million records) and SpeciesLink (http://splink.cria.org.br/; 300,000 records) were retrieved in May 2014. We complemented these two large datasets with all collection records from the 2015 institutional databases of three primarily Amazonian herbaria (INPA, MG, and COAH; 170,000; 180,000, and 28,000 records respectively). We also included a previously compiled dataset of plant collections in all herbaria in Guyana, Suriname, and French Guiana, containing data from these three countries; at the time of the study these records had not been added to GBIF^22^ (190,000 records). All families and genera that do not contain any tree species were removed. A tree was defined as a species with a woody trunk of at least 10 cm diameter at breast height (DBH, where breast height = 1.30 m). Tree-like species that may have a trunk but that are not always taken into account when establishing tree plots (e.g., tree ferns, *Montrichardia*, *Phenakospermum*, bamboos, *Zamia*) were removed. Hereafter we refer to this first database as the collections dataset.

In order to determine which names in the collections dataset are considered taxonomically valid tree species, we independently produced a list of all known tree species (including synonyms) for South America, based on three literature sources^11^,^53^,^54^. We checked the spelling and synonymy of all ~56,000 names gathered in this preliminary tree species list with the Taxonomic Name Resolution Service (http://tnrs.iplantcollaborative.org/TNRSapp.html), the Plant List (http://www.theplantlist.org/), and the Brazilian flora checklist (http://reflora.jbrj.gov.br), to achieve a standard taxonomy (e.g., APG3 plant families) and yield a list of all South American trees synchronized with the ATDN (http://atdn.myspecies.info/) plot database^11^. This required the merging of *Crepidospermum* and *Tetragastris* into *Protium*^50^. Hereafter we refer to this second database as the South American trees reference list, or reference list.

All names in the collections dataset were compared with the corrected names in the South American trees reference list. Species in the collections dataset that matched a species in the reference list were considered a tree species with a valid name or a proper synonym and added to a third database, which is the preliminary checklist of Amazonian trees we present in Appendix S1 (hereafter the checklist). Species in the collections dataset that did not match any species in the reference list were checked against TNRS and then re-matched with the reference list. After this automated procedure, remaining problems were assessed one by one and solved by literature search and with the Plantminer (http://www.plantminer.com/), which mines various sources through the Plant List. If after inspection some of the unmatched tree species names in the collections dataset were indeed correct, they were added to the reference list and to the checklist. All doubtful names (unresolved, illegitimate, invalid) were checked against the original sources where possible and added to the checklist if they had been published correctly and removed if not. Annonaceae names were standardized to the most recent index to this family^15^ and *Guatteria* was standardized to the most recent monograph of the genus^16^.

In order to keep only the Amazonian collections in our collections dataset, we checked all coordinates, corrected them where possible, and removed doubtful coordinates. We accepted as Amazonian all collections located inside Amazonia^11^, as well as all collections without coordinates, but arguably made inside Amazonia based on country (Guyana, Suriname, French Guiana, all collections) or departments that are fully or mainly Amazonian (e.g. Pará, Rondônia, Roraima and Amapá, in Brazil; Amazonas, in all countries).

At this point, the new collections dataset consisted of all Amazonian herbarium records identified to a valid species name. However, because a given herbarium record may occur in GBIF, SpeciesLink, and our herbarium databases, our collections dataset contained a large number of duplicate records. To select unique collections we pulled out all unique combinations of species, collection number, year, country, and province. While this may have omitted some collections that lack a collection number, we believe this number is relatively small. Importantly, the method retained all species names in the data.

During the analyses we noticed that several tree species listed in recent checklists^22^,^54^ and catalogues of Ecuador, Peru and Bolivia (www.tropicos.org) were not in our South American trees reference list. We added these species after checking the names and locations as described above. Finally, we consulted the International Plant Name Index (http://www.ipni.org/) for all species that were newly added to IPNI from 1990 to 2015 in the nine Amazonian countries (9998 species) and added those tree species that we could reliably attribute to Amazonia. The checklists and new species from IPNI added species with no occurrence records to our checklist. For these and species with very few records (together ~1000 species) we searched GBIF once more on a species-by-species basis and added unique record locations within Amazonia. Species for which we found no collection or plot data were added to the checklist but were not included in the numerical analyses, except in the analysis to compare range sizes with the number of collections.

### Data analysis

For species confirmed to occur in the Amazon Tree Diversity Network (ATDN) of 1170 tree plots established across the region, we included in the checklist how many plots it occurred in, how many stems occur in those plots, and estimated Amazonian population size^11^ (see Appendix S1). We used simple regression to test the effect of a species’ estimated population size and range size (which we here defined as the number of ATDN tree plots in which a species was found) on the number of times it had been collected and the earliest date it was collected in Amazonia, to test the hypothesis that more abundant and widespread species are collected more often and earlier than rarer species. Finally we produced a list of all unique collections by country to serve as country checklists. For these lists we only used the collection data. All tests and graphing the data were carried out with custom scripts in R^76^.

### Maps

All maps were created with custom scripts in R^76^. The geographic information (country.shp and river.shp) are shape files from ESRI (http://www.esri.com/data/basemaps, © Esri, DeLorme Publishing Company).

## Additional Information

**How to cite this article**: ter Steege, H. *et al.* The discovery of the Amazonian tree flora with an updated checklist of all known tree taxa. *Sci. Rep.* **6**, 29549; doi: 10.1038/srep29549 (2016).

## Supplementary Material

Supplementary Information

Supplementary Dataset 1

## Figures and Tables

**Table 1:** Collection data by country.

country	^#^collections	grid cells	Area (km^2^)	collections/100 km^2^	^#^species collected	^#^species estimated	%species collected
Bolivia	11721	40	492840	2.38	2404	6060	40
Brazil	278165	333	4102893	6.78	7694	12655	61
Colombia	35277	41	505161	6.98	3511	10073	35
Ecuador	16202	9	110889	14.61	2366	6827	35
French Guiana	64762	7	86247	75.09	2303	*	*
Guyana	36445	15	184815	19.72	2820	*	*
Peru	39851	62	763902	5.22	4422	9336	47
Suriname	31246	11	135531	23.05	2030	*	*
Venezuela	16356	38	468198	3.49	3189	6563	49
**total**	**530025**	**556**	**6850476**	**7.74**	**11194**	**16000**	**70**
*Guianas	132453	33	406593	32.58	3744	4581	82

Number of collections; Number of grid cells; Area of country based on grid cells (km^2^); Collection density (collections/100 km^2^); Number of species collected; Number of species collected based on ref. 11; Collection completeness as the percentage species collected of those expected. The latter two are combined for the three Guianas separate species estimate was made^11^.
