# Supplementary material for: The discovery of the Amazonian tree flora with an updated checklist of all known tree taxa
Source: Sci Rep. 2016 Jul 13;6:29549. doi: 10.1038/srep29549 (PMC4942782; doi:10.1038/srep29549)
Supplement: Supplementary Information [file srep29549-s1.pdf]

Supplemental Material

for

The discovery of the Amazonian tree flora with an updated checklist of all known tree taxa

Hans ter Steege[1,2*], Rens W. Vaessen[1,3], Dairon Cárdenas-López[4], Daniel Sabatier[5], Alexandre Antonelli[6,7], Sylvia Mota de Oliveira[1], Nigel C.A. Pitman[8,9], Peter Møller Jørgensen[10], Rafael P. Salomão[2]

1 Naturalis Biodiversity Center, Vondellaan 55, Postbus 9517, 2300 RA Leiden, The Netherlands

2 Coordenação de Botânica, Museu Paraense Emílio Goeldi, Av. Magalhães Barata 376, C.P. 399, Belém, PA 66040–170, Brazil

3 current address: Ecology and Biodiversity Group, Utrecht University, Padualaan 8, 3584 CH Utrecht, The Netherlands

4 Herbario Amazónico Colombiano, Instituto SINCHI, Calle 20 No 5-44, Bogotá, DF, Colombia

5 Institut de Recherche pour le Développement (IRD, UMR AMAP), TA A-51/PS2, Bd. de la Lironde, 34398 Montpellier cedex 5, France

6 Department of Biological and Environmental Sciences, University of Gothenburg, Box 461, SE-405 30 Göteborg, Sweden

7 Gothenburg Botanical Garden, Carl Skottsbergs gata 22A, SE-413 19, Göteborg, Sweden

8 Science and Education, The Field Museum, 1400 S. Lake Shore Drive, Chicago, IL 60605–2496, USA

9 Nicholas School of the Environment, Duke University, Durham, North Carolina 27705, USA

10 Missouri Botanical Garden, P.O. Box 299, St. Louis, Missouri 63166-0299, USA

* Corresponding author. Email: hans.tersteege@naturalis.nl

Table S1. Number of herbarium specimens of Amazonian tree species, collection density, and the most prolific collectors for the nine Amazonian countries, ordered by collection records/100km^2.

Country	Records	Records/100 km^2	Main collectors in the 20th century
French Guiana	64,762	75	D. Sabatier, J.-J. de Granville, R.A.A. Oldeman, S.A. Mori, M.F. Prévost, G. Cremers, D. Loubry, P. Grenand, J.F. Molino, J.F. Villiers, C.H.L Sastre, B.M. Boom, C. Feuillet
Suriname	31,246	23	J.C. Lindeman, J. Lanjouw, J. Van Donselaar, B. Maguire, G. Stahel, R. Evans
Guyana	36,445	20	H.D. Clarke, M. Jansen-Jacobs, D.B. Fanshawe, G.S. Jenman, J. Pipoly, T. Henkel, B. Hoffman, T. McDowell, L.J. Gillespie, T.R. van Andel, P.J.M. Maas
Ecuador	16,202	15	C.A. Cerón, A. Dik, D. Neill, H. Balslev, W. Palacios, B. Øllgaard, A.H. Gentry, J. Brandbyge, E. Gudiño
Colombia	35,227	7.0	D. Cardenas, A. Rudas, M. Sánchez, R. López, H. Mendoza, A. Eusse, L.E. Urrego, M.P. Cordoba, A. Duque, Julio Betancur, Nicolas Castaño
Brazil	278,165	6.8	G.T. Prance, C.F. da Silva, W.A. Rodrigues, C.I.D. Ferreira, A.W. Ducke, D. Daly, J. Murça Pires, B.A. Krukoff, R. Souza
Peru	39,851	5.2	A.H. Gentry, R. Vásquez, R. Foster
Venezuela	16,356	3.5	M. Angel, J.A. Steyermark, O. Huber, B. Maguire, B.M. Boom, A.H. Gentry, R.L. Liesner, G.A. Aymard
Bolivia	11,721	2.4	M. Nee, J. Guillen, T. Killeen, A. Jardim, G. James, G.T. Prance

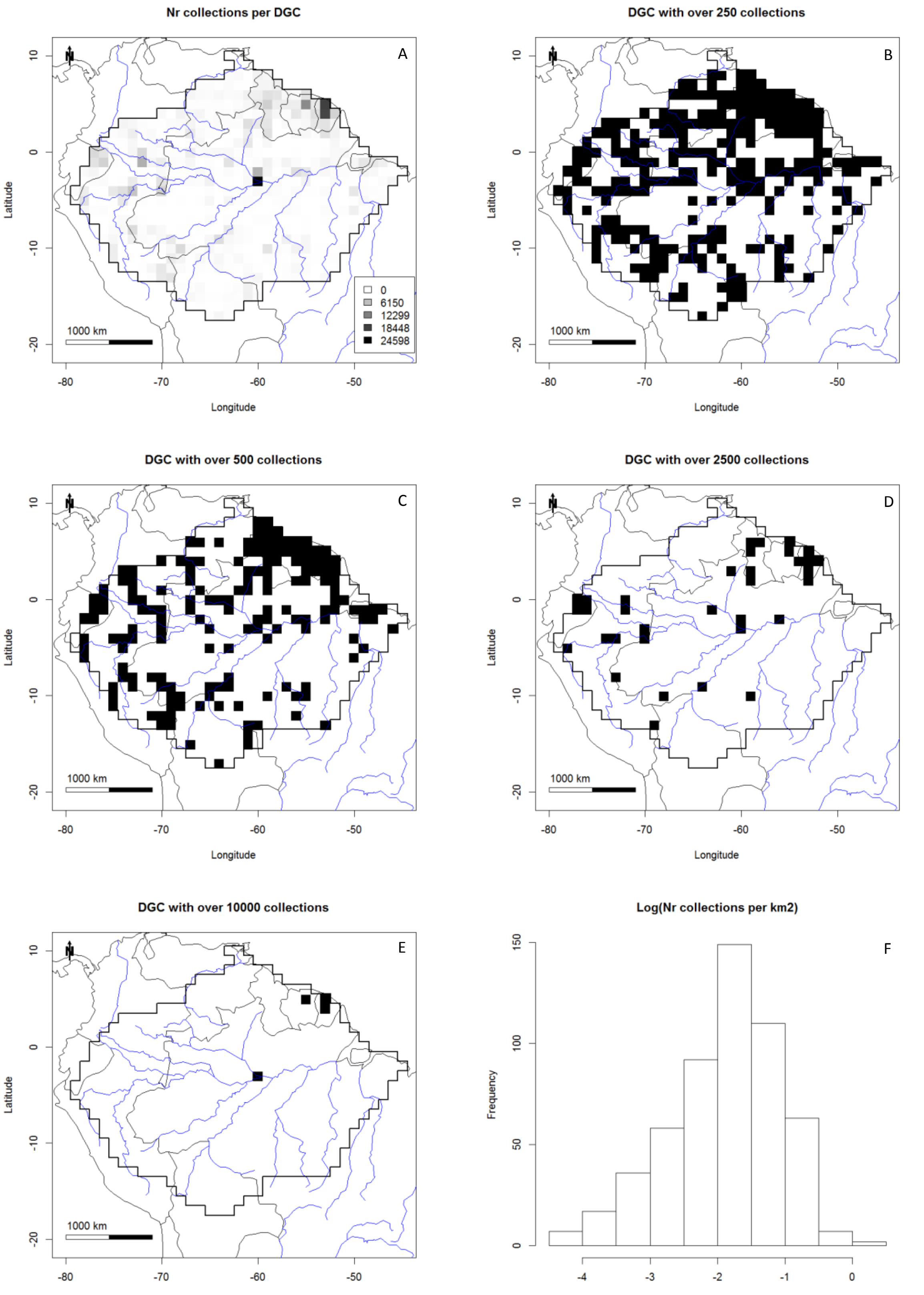

Figure S1

Figure S1. **A**. Average number of collections of trees per 1 degree grid cell (DGC). **B**. DGSs with over 250 collections. **C**. DGSs with over 500 collections. **D**. DGSs with over 2,500 collections. **E**. DGSs with over 10,000 collections. **F**. Histogram of log(number of collections) by DGC. Maps created with custom R script. Base map source (country.shp, rivers.shp): ESRI (http://www.esri.com/data/basemaps, © Esri, DeLorme Publishing Company).

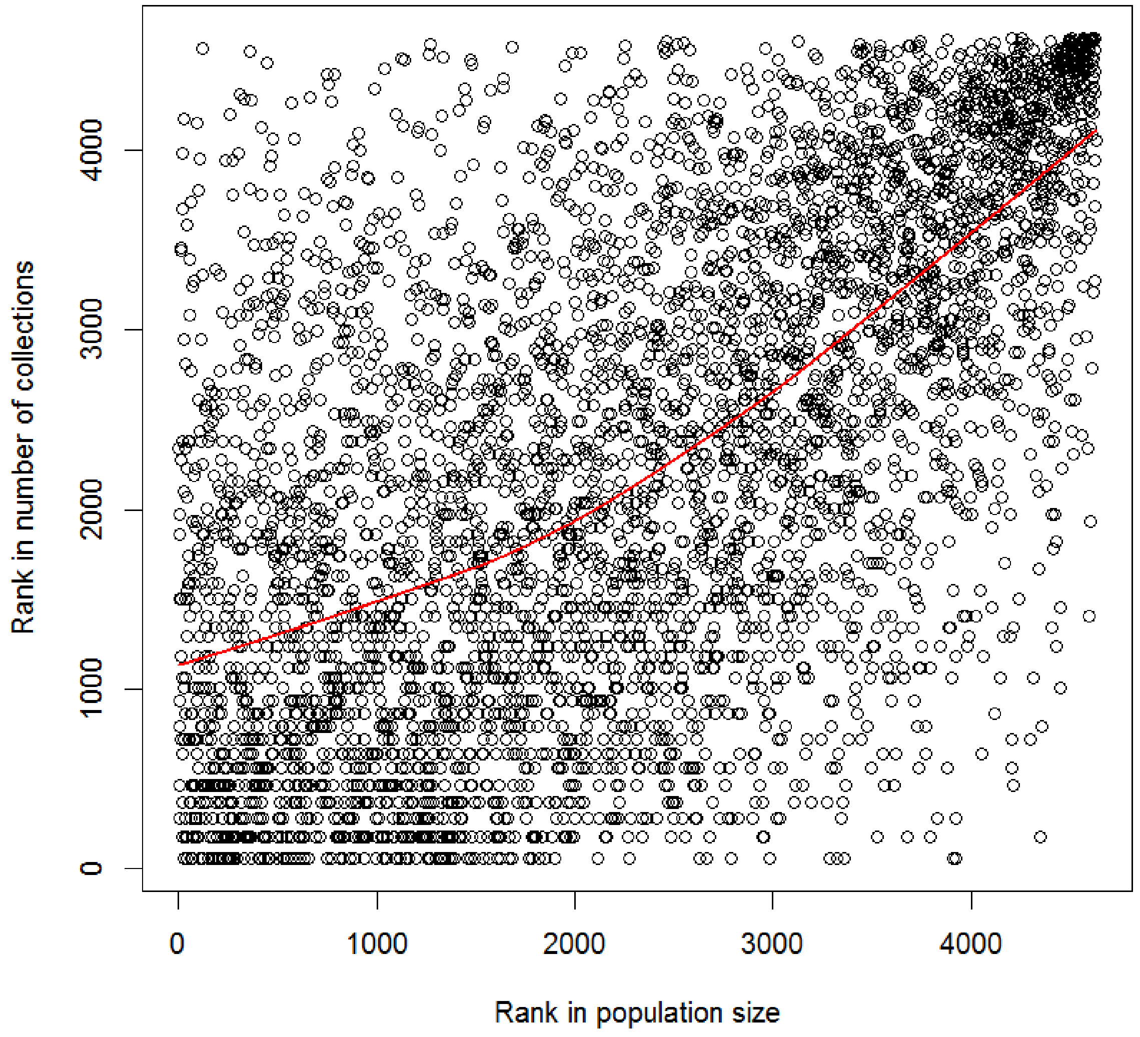

Figure S2. Rank in number of collections as a function of the rank in estimated basin-wide population size for 4523 Amazonian tree species.

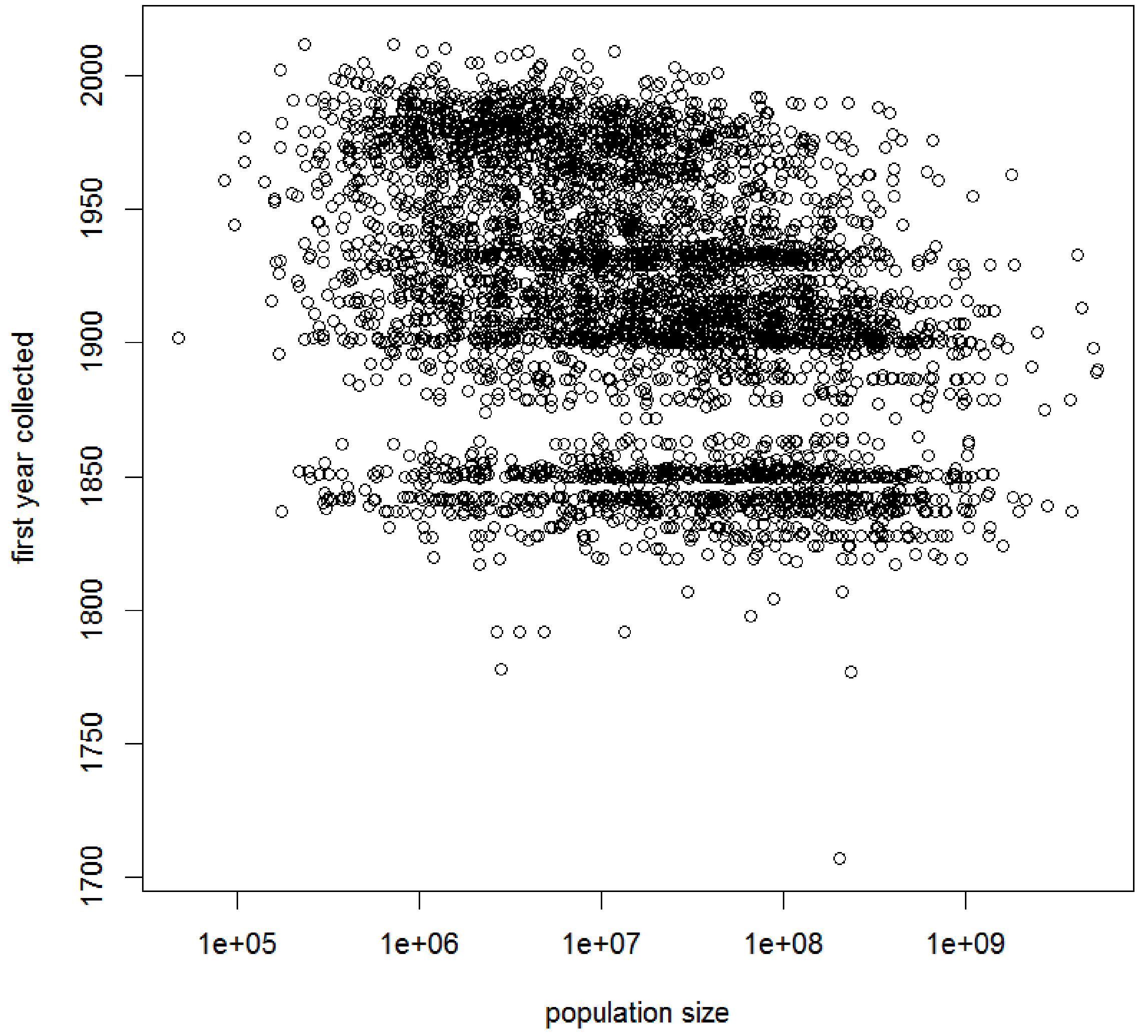

Figure S3. The year in which a tree species was first collected in Amazonia as a function of its estimated basin-wide population size.

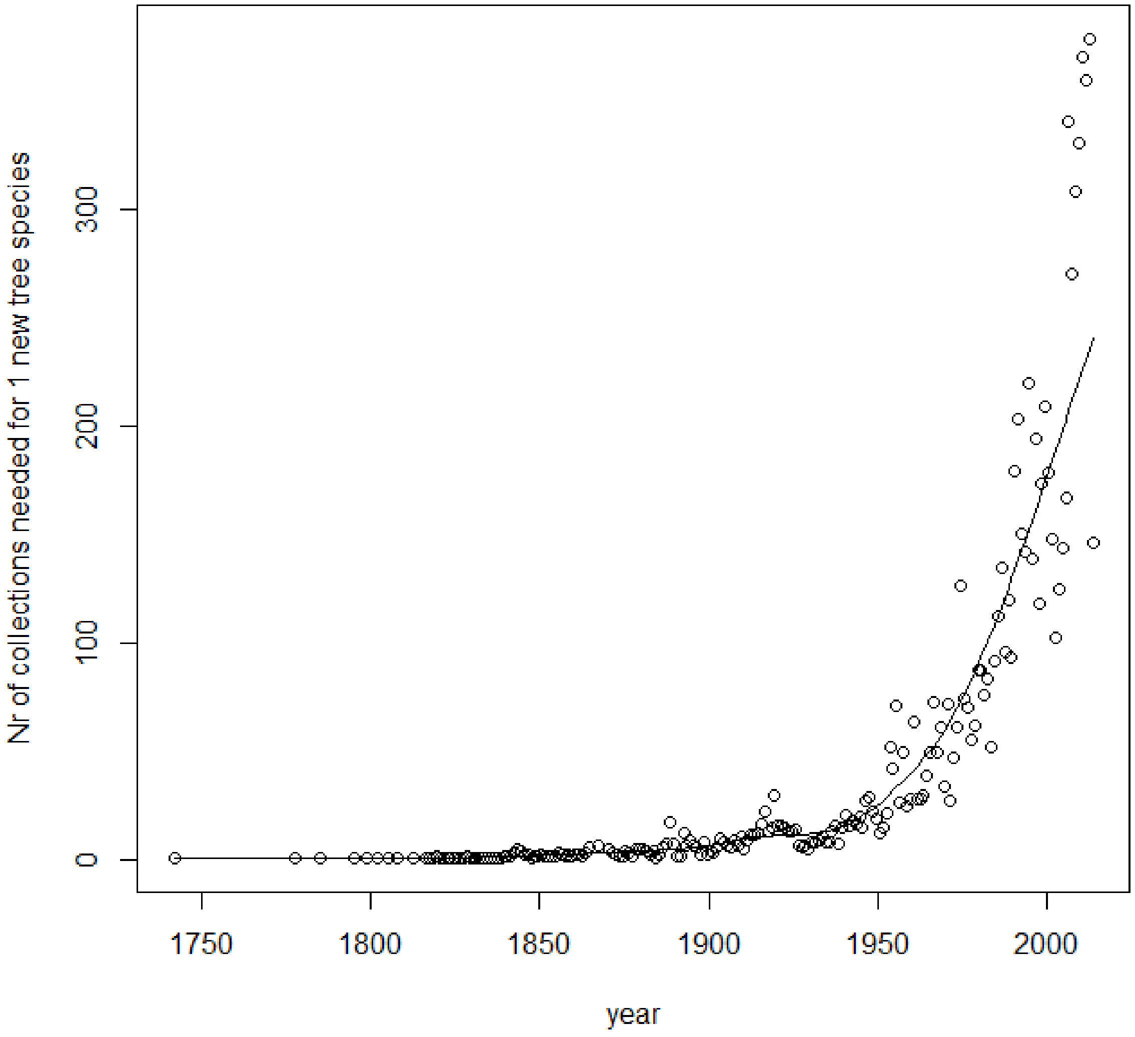

Figure S4. The number of collections of Amazonian trees required to discover one new species each year, from 1707 to 2015.

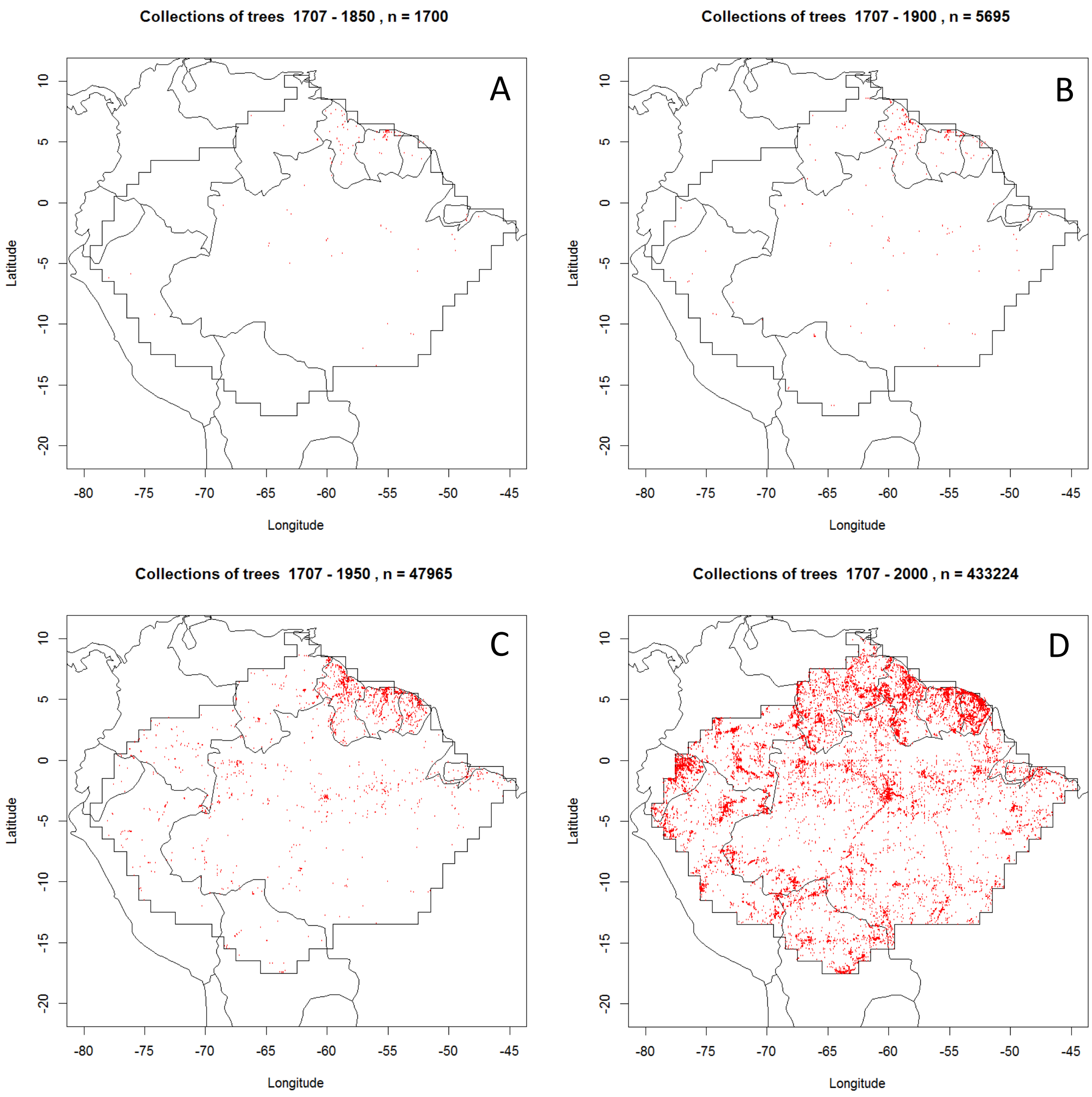

Figure S5. Collection localities of all Amazonian tree specimens in herbarium databases for which geographical coordinates were available and considered reliable. **A**. period 1707 – 1850. **B**. period 1707 – 1900. **C**. period 1707 – 1950. **D**. period 1707 – 2000. Maps created with custom R script. Base map source (country.shp, rivers.shp): ESRI (http://www.esri.com/data/basemaps, © Esri, DeLorme Publishing Company).

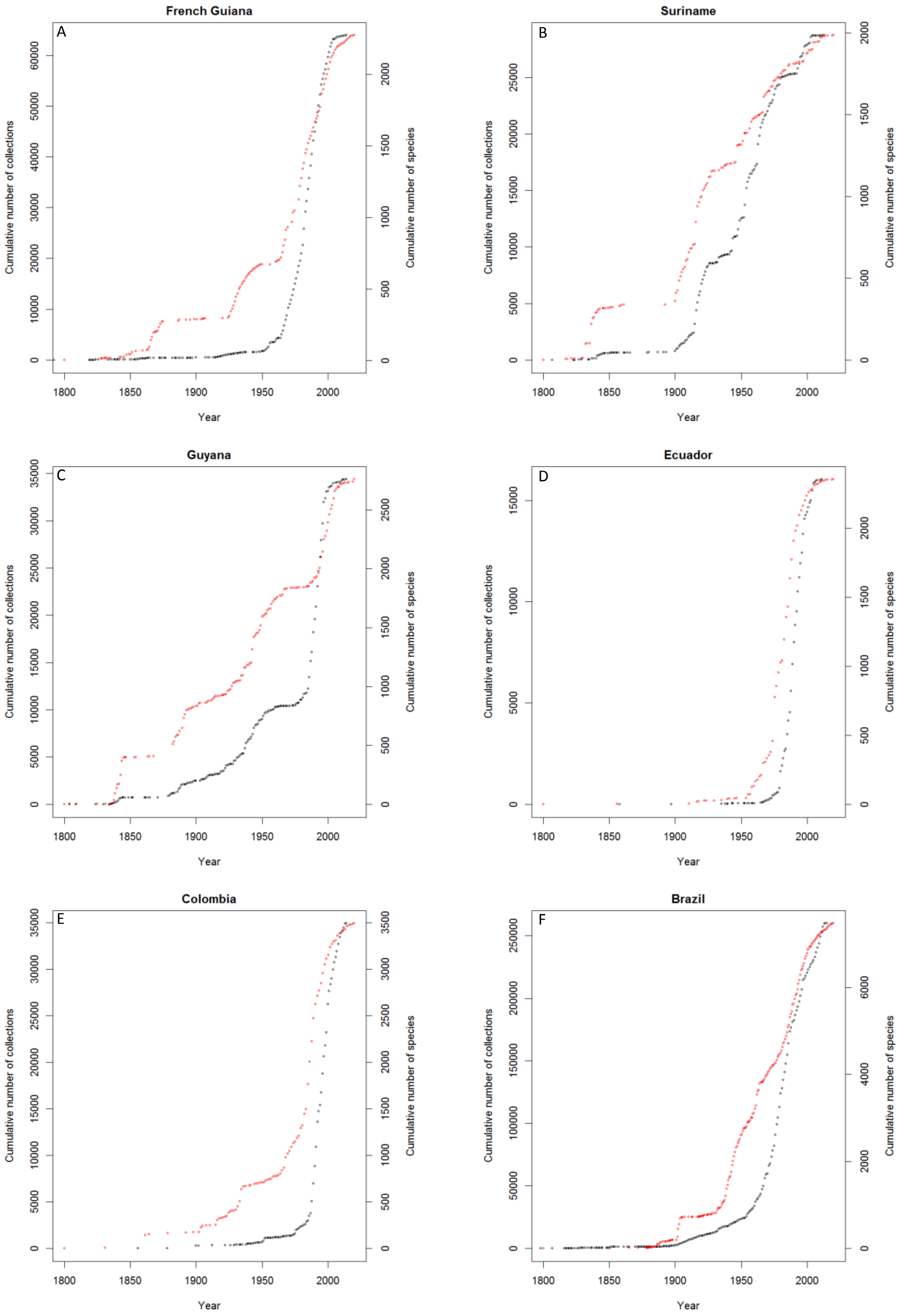

French Guiana
A
Suriname
B
Guyana
C
Ecuador
D
Colombia
E
Brazil
F
Cumulative number of collections
Cumulative number of species
Year
1800
1850
1900
1950
2000

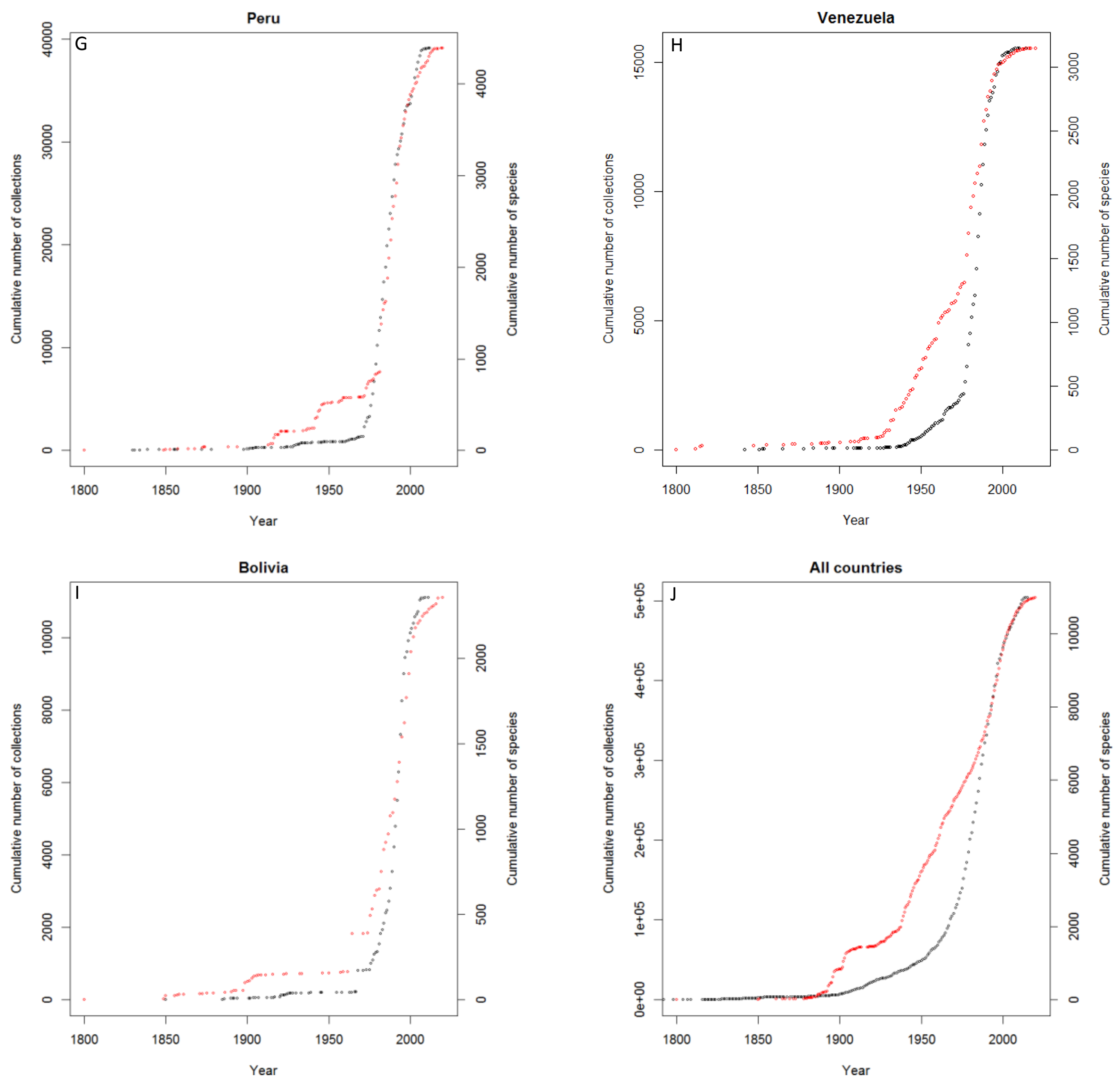

Figure S6. Cumulative number of tree species collections in Amazonia shown from 1800 to 2015 (Black line, left y-axis) and cumulative number of species collected from 1800 to 2015 (red line, right y-axis). **A**. French Guiana, **B**. Suriname. **C**. Guyana, **D**. Ecuador, **E**. Colombia, **F**. Brazil, **G**. Peru, **H.** Venezuela, **I**. Bolivia, J. All countries together.

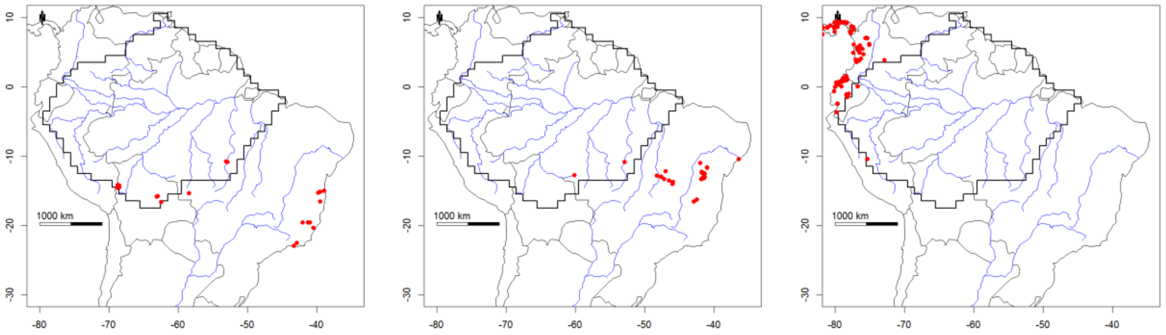

Figure S7. Examples of ‘non-Amazonian’ tree species with few records in Amazonia. Maps with GBIF records of *Cariniana ianeirensis* (an Atlantic forest species), *Exellodendron gardneri* (a caatinga species) and *Conostegia montana* (a montane species). Maps created with custom R script. Base map source (country.shp, rivers.shp): ESRI (http://www.esri.com/data/basemaps, © Esri, DeLorme Publishing Company).

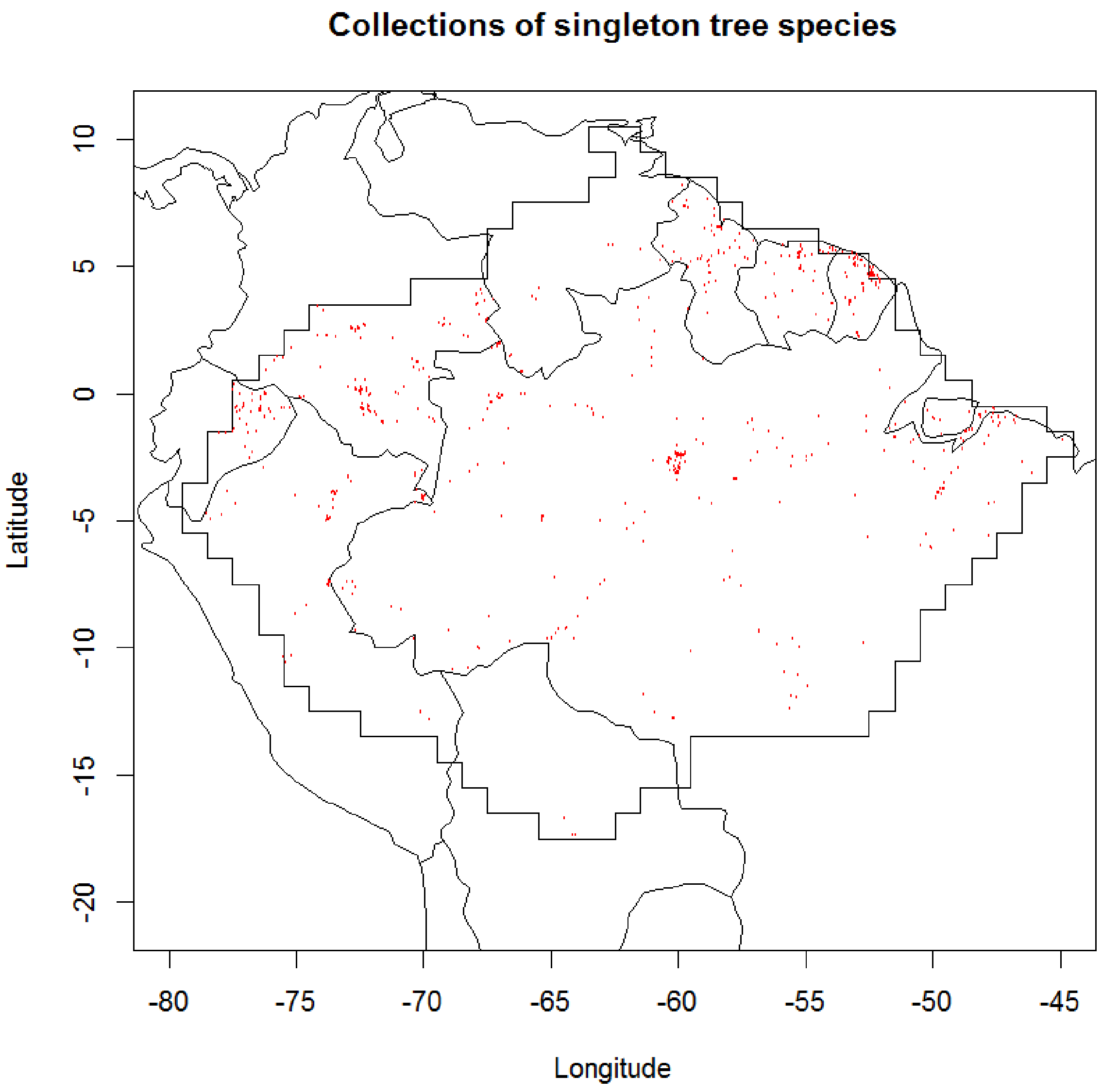

Figure S8. Collection locations of singleton species in the collection data. Most singletons are found in areas that are well collected. Map created with custom R script. Base map source (country.shp, rivers.shp): ESRI (http://www.esri.com/data/basemaps, © Esri, DeLorme Publishing Company).

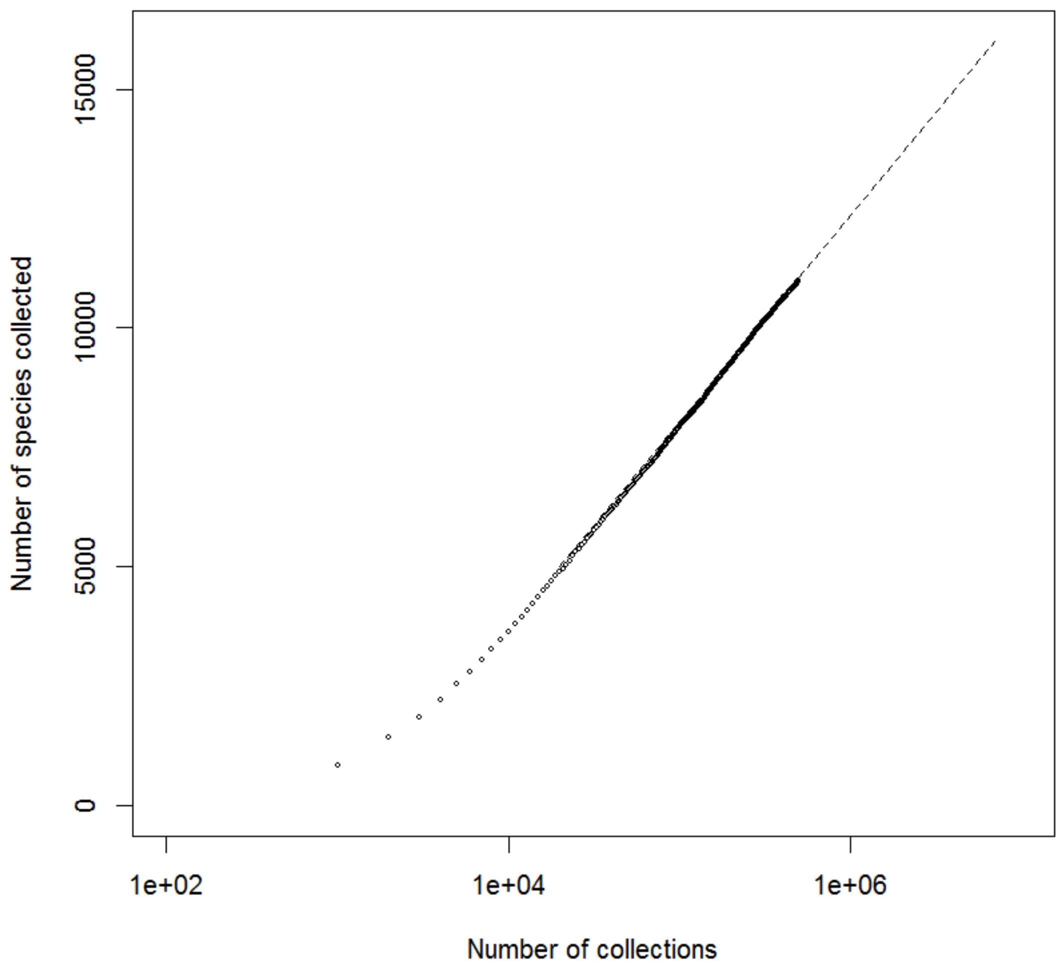

Figure S9. Cumulative number of Amazonian tree species as a function of the number of collections (logarithmic axis). A lognormal extrapolation predicts that ~5,000,000 collections will be required to record 16,000 species.

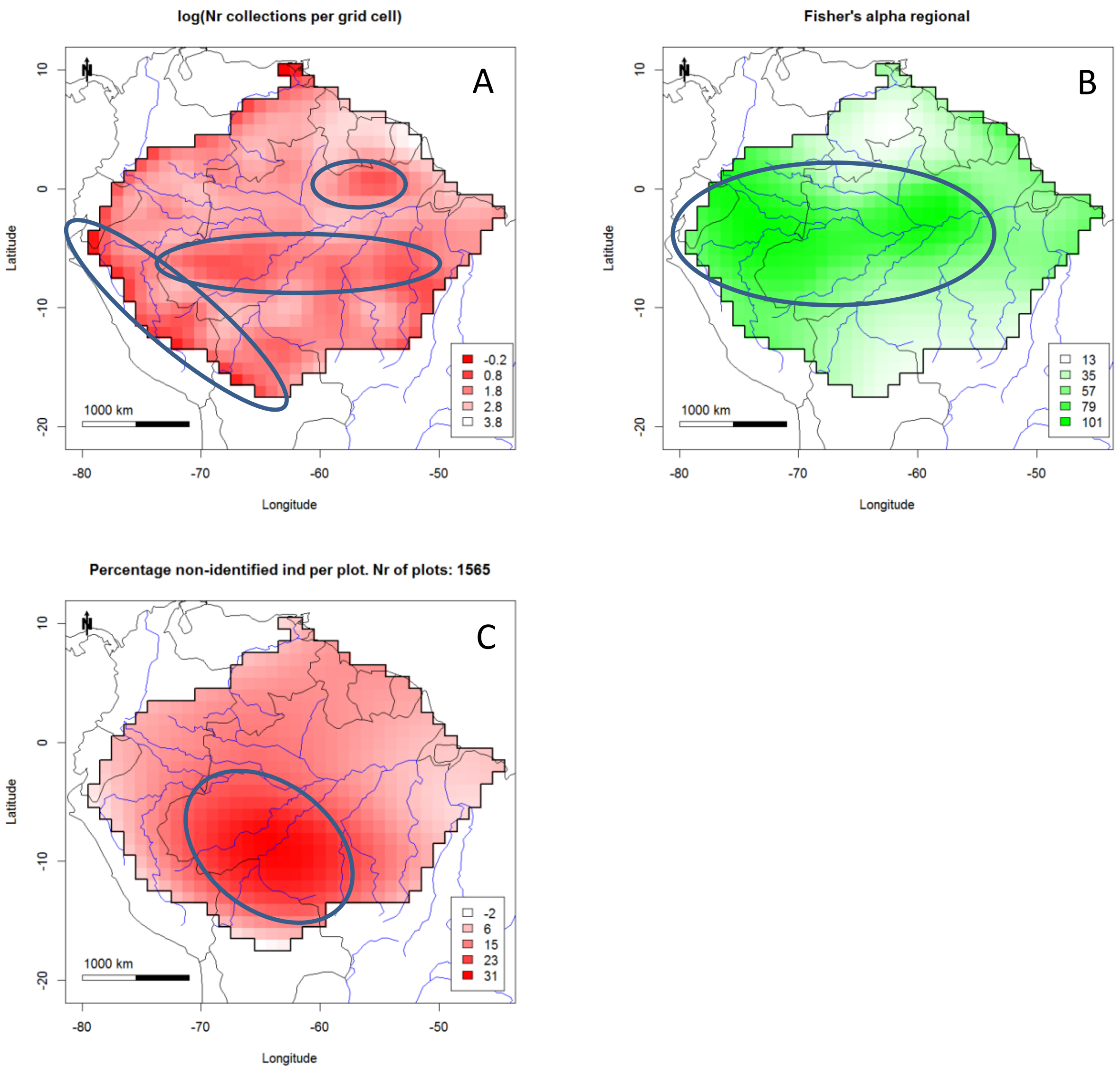

Figure S10. **A**. Map of the logarithm of average number of collections of trees per 1 degree grid cell (DGC). **B**. Map of average Fisher's α by DGC (courtesy Amazon Tree Diversity Network). **C**. Map of Interpolated percentage of species-level taxa not identified to species in tree inventory plots in Amazonia[1]. Maps created with custom R script. Base map source (country.shp, rivers.shp): ESRI (http://www.esri.com/data/basemaps, © Esri, DeLorme Publishing Company).

Legend to Appendices

S1. Species names of 11,676 trees recorded to date in Amazonia. Species, Author: species name and authority as reported by source (see field Source Name); collections: number of collections found in the herbarium sources consulted; n.dgc.coll: number of 1 degree grid cells for which the species has valid geo-information; n.ind: number of individuals observed in 1170 plots in Amazonia[2]; n.plots: number of plots in which the species has been observed, of 1170 plots in Amazonia[2]; population.mean: estimated population in Amazonia[2]; FirstCollyy: First year of collection in Amazonia based on herbarium data consulted; Family: botanical family of species (APG3); Source Name: data source for name; url: url of accepted name; TreeSource: data source for trees (ATDN: species found as tree > 10 cm DBH in ATDN plots [2]; Aublet: y if species was collected by Aublet (211 species); note: additional notes.

S2. **Species checklists by countries**. Total number of unique collections found in the primary databases of GBIF, SpeciesLink, MG, INPA, COAH by country. Last column numbers for the three Guianas together.

S3. A. Number of species and genera by family. **B.** Number of species by genus.

References

1 ter Steege, H. *et al.* Estimating the global conservation status of over 15,000 Amazonian tree species. *Science Advances* **1**, e1500936, doi:10.1126/sciadv.1500936 (2015).

2 ter Steege, H. *et al.* Hyperdominance in the Amazonian Tree Flora. *Science* **342**, 1243092, doi:10.1126/science.1243092 (2013).

Data providers for primary tree data (number of collections of trees).

A (55), Acalypha (184), ALTA-VP (1), ALTA-VP COLLECTION (1), ARIZ (9), Aublet (17093), BOG (3), BOT (988), Botany (21413), BRIT (3209), BSM plantscoll (1), BW Suriname (58), Cenargen (2415), CITSF (7), CNPC (48), COAH (24986), COL (2846), CONN (43), CPAP (73), CPATSA (85), CPQBA (21), CVRD (84), DS (1), E (89), EBDA (30), ECON (5), ESALQ (2623), F (2666), FEMACT (2355), Field Museum (40), FMB (7916), FSL (1182), Fundacion Puerto Rastrojo (2233), FURB (101), FZB-BH (8), GBIF (133), GH (74), HERB (42), Herbario Nacional de Costa Rica (CR) (1), Herbarium (30141), Herbarium Berolinense (47), Herbarium Senckenbergianum (157), Herbarium University Ulm (117), Herbarium W (34), Herbarium WU (248), Herbier de la Guyane (96889), HPL (353), HUA (402), HUCO (13), IAC (956), IAvH-CT (194), IBt (2521), IECOS (1139), IF (171), IFAM - CMZL (714), IMA (320), INPA (145856), Institut de Recherche pour le Developpement (IRD) (23), Instituto de Botanica (1), IPA (1072), IPT (4105), Iwokrama (115), JBB (296), JBRJ (27377), Jenman Herbarium (65), Jonah Boyan Herbarium (638), KANU (2), L (11225), LBB (7), Lindeman (16074), Lynn Gillespie (52), MA (137), MADAM (4), MBM (2619), MBML (12), MCN/FZBRS (7), MCN-PUC-MINAS (2), MEXU (19), MG (27400), MN (1707), MO (722), MPEG (38906), MPU (3), Museu Botanico Municipal Curritiba, Brasilia (1), NA (22434), NCY (11), NSW (13), NYBG (68542), P (2602), PalmTransect (342320), PNFM (34), PUCRS (16), RB (9340), S-Vascular (222), THETIS-IBN (3), Tropicos (161993), U (42530), UBC (70), UDESC (4), UEFS (1728), UEL (191), UEM (12), UEMA (742), UEPA (2553), UESB (8), UFAC (135), UFBA (119), UFC (581), UFERSA (6), UFES (5), UFG (151), UFJF (73), UFMA (233), UFMG (384), UFMS (161), UFMT (530), UFOP (3), UFPA (164), UFPB (133), UFPE (64), UFPI (96), UFPR (700), UFRGS (175), UFRN (69), UFRPE (287), UFRR (433), UFS (24), UFSC (186), UFSCar (28), UFSJ (26), UFU (49), UFV (141), UFVJM (64), UNA (5), UnB (4622), UNEMAT (3913), UNESC (2), UNESPFCA (88), UNESPIBB (63), UNESPRC (322), UNESPSJRP (47), UNICAMP (2436), UNICAP (51), UNICENTRO (1), UNIR (4811), UNISANTA (4), UNITINS (78), UNIVASF (6), University of Suriname (1), UNIVILLE (4), URCA (47), URICER (3), US (7335), USC (19), USP (1157), UTC (1), UTFPR (65), Veth (4), ZMT (11), ZMT/UABCS (3).
